# Musclin, A Myokine Induced by Aerobic Exercise, Retards Muscle Atrophy During Cancer Cachexia in Mice

**DOI:** 10.3390/cancers11101541

**Published:** 2019-10-12

**Authors:** Andrea D. Re Cecconi, Mara Forti, Michela Chiappa, Zhiyong Zhu, Leonid V. Zingman, Luigi Cervo, Luca Beltrame, Sergio Marchini, Rosanna Piccirillo

**Affiliations:** 1 Department of Neurosciences, Mario Negri Institute for Pharmacological Research IRCCS, 20156 Milan, Italy; andrea.rececconi@marionegri.it (A.D.R.C.); mara.forti@guest.marionegri.it (M.F.); michela.chiappa@marionegri.it (M.C.); luigi.cervo@marionegri.it (L.C.); 2 Department of Internal Medicine, University of Iowa Carver College of Medicine, Iowa City, IA 52242, USA; zhiyong-zhu@uiowa.edu (Z.Z.); leonid-zingman@uiowa.edu (L.V.Z.); 3 Fraternal Order of Eagles Diabetes Research Center, University of Iowa Carver College of Medicine, Iowa City, IA 52242, USA; 4 Department of Medicine, Medical Center, Department of Veterans Affairs, Iowa City, IA 52246, USA; 5 Department of Oncology, Mario Negri Institute for Pharmacological Research IRCCS, 20156 Milan, Italy; luca.beltrame@marionegri.it (L.B.); sergio.marchini@marionegri.it (S.M.)

**Keywords:** muscle wasting, muscle atrophy, aerobic exercise, cancer cachexia, myokine, musclin, PGC1α

## Abstract

Physical activity improves the prognosis of cancer patients, partly by contrasting the associated muscle wasting (cachexia), through still unknown mechanisms. We asked whether aerobic exercise causes secretion by skeletal muscles of proteins (myokines) that may contrast cachexia. Media conditioned by peroxisome proliferator-activated receptor γ coactivator 1α (PGC1α)-expressing myotubes, reproducing some metabolic adaptations of aerobic exercise, as increased mitochondrial biogenesis and oxidative phosphorylation, restrained constitutively active Forkhead box-containing subfamily O3 (caFoxO3)-induced proteolysis. Microarray analysis identified amphiregulin (AREG), natriuretic peptide precursor B (NppB), musclin and fibroblast growth factor 18 (FGF18) as myokines highly induced by PGC1α. Notably, only musclin tended to be low in muscle of mice with a rare human renal carcinoma; it was reduced in plasma and in muscles of C26-bearing mice and in atrophying myotubes, where PGC1α expression is impaired. Therefore, we electroporated the Tibialis Anterior (TA) of C26-bearing mice with musclin or (its receptor) natriuretic peptide receptor 3 (Npr3)-encoding plasmids and found a preserved fiber area, as a result of restrained proteolysis. Musclin knockout (KO) mice lose more muscle tissue during growth of two distinct cachexia-causing tumors. Running protected C26-bearing mice from cachexia, not changing tumor growth, and rescued the C26-induced downregulation of musclin in muscles and plasma. Musclin expression did not change in overloaded plantaris of mice, recapitulating partially muscle adaptations to anaerobic exercise. Musclin might, therefore, be beneficial to cancer patients who cannot exercise and are at risk of cachexia and may help to explain how aerobic exercise alleviates cancer-induced muscle wasting.

## 1. Introduction

Cachexia is estimated to occur in about 85% of patients with diverse types of cancer and leads to death in about 25% of them, through massive loss of adipose tissue and muscle mass [1,2]. During cachexia, systemic inflammation increases through elevated levels of pro-inflammatory cytokines, such as interleukin 6 (IL6) or tumor necrosis factor α (TNFα) [3]. The main transcription factor driving the expression of muscle-restricted ubiquitin ligases (atrogin-1 and Muscle RING-finger protein-1 or MuRF1), ultimately enhancing proteasomal protein breakdown in muscle, is Forkhead box-containing subfamily O3 (FoxO3) [4]. Its activation is promoted also by pro-inflammatory molecules [5]. Unfortunately, anti-inflammatory therapies have limited anti-cachexia effects and circulating biomarker(s) to detect the syndrome are largely unknown [6], but they would be very useful to treat on time cancer patients at risk of developing cachexia.

Physical exercise can contrast cachexia in cancer patients, by reducing systemic inflammation [7], increasing muscle mass and/or switching fiber type [8]. Physical activity can be classified as anaerobic (or strength) or aerobic (or endurance) exercise. In the former, activation of the Ser/Thr kinase AKT enhances protein synthesis [9], ultimately causing hypertrophy especially of glycolytic fibers (type II fibers) [10]. In the latter, AMP-activated protein kinase (AMPK)-driven activation of the peroxisome proliferator-activated receptor γ coactivator 1α (PGC1α) in rodents [11] and in humans [12] favors the shift of fibers towards oxidative type I fibers, by promoting mitochondrial biogenesis [13], with no appreciable increase in muscle mass [14]. Oxidative fibers are more resistant than glycolytic ones to various types of atrophy, including that induced by cancer [15]. PGC1α counteracts muscle atrophy in mice [16,17], directly antagonizing FoxO3 binding to DNA [16], while AKT inactivates FoxO3 by phosphorylating it [4].

In recent years, skeletal muscle has been shown to secrete molecules (myokines) that can act at short and long distance on muscle itself or other tissues, with an impact on overall body metabolism [18]. Some of them are induced by specific types of exercise [19]. The PGC1α-induced myokine irisin is the cleaved soluble form of fibronectin type III domain-containing protein 5 (FNDC5) and engaged in brown-to-white fat conversion [20]. Fibroblast Growth Factor 21 (FGF21) is induced by AKT and involved in the metabolic response to fasting [21]. Aerobic exercise seems more powerful than anaerobic in preserving muscle from wasting during cancer [22], in fact, the AKT/mammalian target of rapamycin (mTOR) signaling appears even hyperactivated in muscles of cancer-bearing rodents [23]. We therefore asked whether PGC1α promotes the secretion of myokines that may contrast cancer-associated muscle atrophy.

In this work, we identified PGC1α-induced myokines and found that only the expression of musclin, unlike that of its family member, natriuretic peptide precursor B (NppB), is almost abrogated in the muscles of colon adenocarcinoma C26-bearing mice. We also show that overexpression of musclin, or its receptor, natriuretic peptide receptor 3 (Npr3), protects muscles from wasting during cancer. Notably, musclin knockout (KO) mice display a slight reduction in overall lean body mass with no gross change in gastrocnemius or quadriceps weights [24] but when we injected them with cachexia-inducing tumors, they lose more muscle tissues and tend to be weaker.

Musclin is an exercise-induced myokine [24,25], initially identified in 2003 as a bone-derived peptide (osteocrin) [26] but expressed in skeletal muscles more than 10-fold than in bone, brown adipose tissues, testis and spleen [26]. Its C-terminal domain is highly homologue to natriuretic peptides (NPs) that are atrial natriuretic peptide (ANP), B-type NP (BNP, mature form of NppB) and C-type NP (CNP) and are involved in fluid homeostasis, cardiac physiology, fat metabolism and skeletal development [27]. Similar to those, musclin is cleaved at an internal serine protease cleavage site [25]. In contrast to the other NPs, musclin is unable to bind Npr1 or 2, but only 3 [28]. While Npr1 and 2 are single membrane-spanning domain proteins, endorsed of guanylate cyclase activity, Npr3 lacks such catalytic activity, possibly acting as a clearance receptor for NPs. Subsequently, musclin peptide may potentiate NP-based signalling. Indeed, musclin seems to function in this way during its suppressive action against heart failure after myocardial infarction in mice [29] or in the modulation of mouse bone growth [30]. Surprisingly, even if these receptors have been found expressed in muscles and myocytes, a possible role of musclin in the control of skeletal muscle size has never been explored in depth.

Our data support the notion that musclin can be induced in skeletal muscles by aerobic exercise and subsequent PGC1α overexpression during C26 tumor growth and this could be added to the mechanisms by which endurance activity alleviates cancer-induced muscle wasting.

## 2. Results

### 2.1. Peroxisome Proliferator-Activated Receptor γ Coactivator 1α (PGC1α)-Overexpressing Myotubes Secrete Unknown Factors with Anti-Catabolic Action

Various studies have shown that aerobic exercise can prevent or alleviate cancer-induced muscle wasting [22]. Since aerobic exercise causes the secretion by skeletal muscles of proteins, namely myokines [31], we asked whether some exercise-induced secretable factors might contrast atrophy in myotubes. To reproduce at least partially in vitro some molecular differences of aerobic muscle exercise that are independent from systemic inflammation or the hormonal milieu, we infected fully differentiated myotubes with adenoviruses expressing PGC1α, one of the main transcriptional coactivators involved in muscle adaptation to endurance exercise [32]. To induce myotube atrophy, we infected cells with constitutively active (ca)FoxO3-expressing adenoviruses, as we and others have already done [33,34].

**Figure 1:**
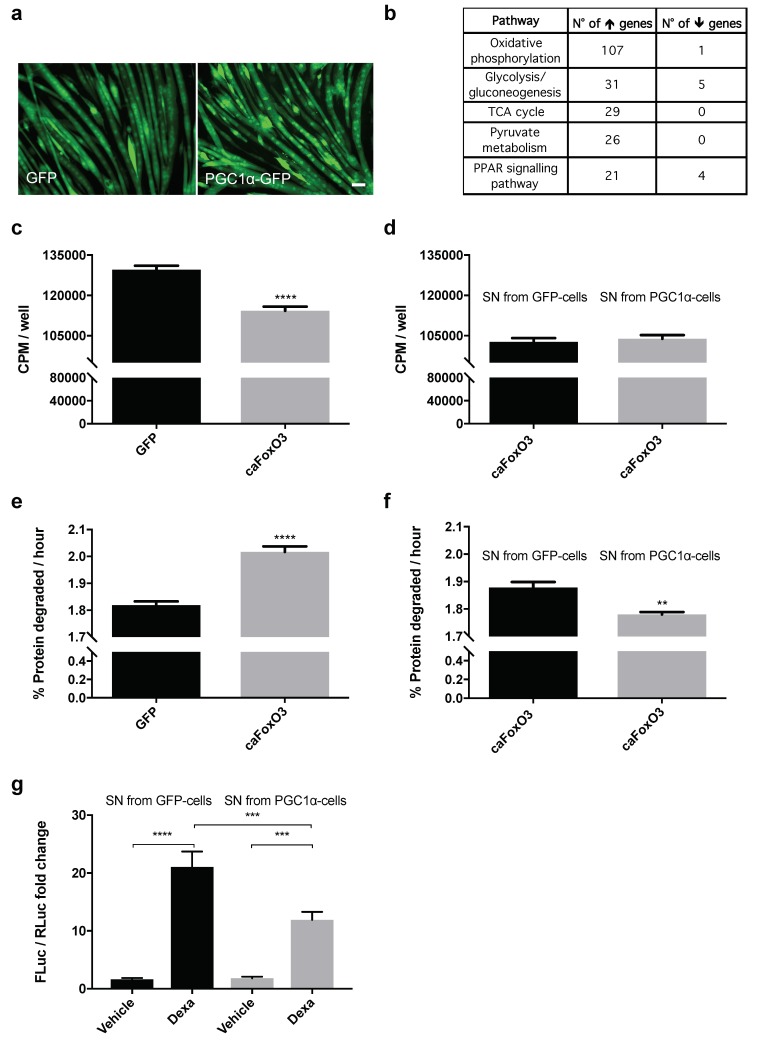
Peroxisome proliferator-activated receptor γ coactivator 1α (PGC1α)-overexpressing myotubes secrete some anti-catabolic factors. Representative fields of four-day differentiated myotubes infected for 48 h with Green Fluorescent Protein (GFP) or PGC1α-GFP-expressing myotubes are shown. Scale bar, 25 μm (**a**). Enriched pathways from Kyoto Encyclopedia of Genes and Genomes (KEGG) (*p* < 0.05) are shown from the microarray differential expression analysis, which involves the mitochondrial function, with the number of upregulated and downregulated genes belonging to each pathway (**b**). Rates of overall protein synthesis and long-lived protein degradation were measured in myotubes transfected on the fourth day of differentiation with GFP or constitutively active Forkhead box-containing subfamily O3 (caFoxO3)-expressing viruses. Infection of myotubes for 48 h with adenoviruses encoding for caFoxO3 reduces protein synthesis (**c**) and increases protein degradation of long-lived proteins (**e**). Unpaired *t*-test, **** *p* ≤ 0.0001, *n* = 6. GFP-expressing adenoviruses are used as control. Supernatants (SN) from myotubes infected for 72 h with PGC1α-encoding adenoviruses do not alter protein synthesis (**d**) but restrain caFoxO3-induced protein degradation (**f**). Unpaired *t*-test, **, *p* ≤ 0.01, *n* = 6. CPM, counts per minute. The dexamethasone-induced MuRF1 promoter-Firefly Luciferase (FLuc) signal is reduced in myoblasts exposed to SN from PGC1α-expressing cells but not from GFP-expressing ones. Myoblasts were transfected with MuRF1 promoter-FLuc plasmid and HSV-thymidine kinase (TK) promoter-Renilla Luc plasmid (RLuc) and 24 h later treated for 24 h with 10 μM dexamethasone and the aforementioned SN. The results of three independent experiments are shown (**g**). One-way analysis of variance (ANOVA) followed by Tukey’s test, *** *p* ≤ 0.001, **** *p* ≤ 0.0001, *n* = 6.

In order to understand how 48 h expression of PGC1α (Figure 1a) could induce changes in myotubes that may mimic partly what aerobic exercise does, we ran microarray analyses on them. Differential expression of PGC1α versus control followed by pathway enrichment (see Methods) identified a number of significant pathways of interest pertaining to mitochondrial functions and metabolism, mostly involving upregulated genes (Figure 1b). PGC1α did not change the total content of RNA (Figure S1a), indirectly suggesting that protein synthesis was not affected as expected, while many genes involved in oxidative phosphorylation were upregulated in these myotubes (Figure 1b). Genes encoding for mitochondrial proteins were also induced by PGC1α in myotubes, such as the mitochondrial ribosomal protein S33, MRPS33 (Figure S1b), confirming PGC1α as a driver of the gene adaptation program typically induced by aerobic exercise.

To assess whether PGC1α promotes the secretion of myokines to contrast atrophy, we used the cell-free supernatant conditioned for 48 h by myotubes infected 24 h earlier with Green Fluorescent Protein (GFP) or PGC1α-expressing adenoviruses. These conditioned media were used for 24 h to treat myotubes atrophying because of caFoxO3 overexpression for 48 h. To avoid transferring any adenoviral particles during supernatant treatment, because we were interested in the effect of secreted molecules and not that of the forced expression of PGC1α on receiving myotubes, we performed four washes with Phosphate-Buffered Saline (PBS) on the cells infected overnight with the viruses. We had found previously that four, not two washes were needed before replacement of the medium to remove any undesirable adenovirus detectable by polymerase chain reaction (PCR) in the supernatant-producing cells (Figure S1c).

As expected, 48 h infection of myotubes with caFoxO3-encoding viruses reduced overall RNA content by about 50% (Figure S1a), overall protein synthesis by 12% (Figure 1c) and increased the rates of long-lived protein degradation per hour by about 11% (Figure 1e), by enhancing both atrogin-1 and MuRF1 expression (Figure S2a–c). The GFP-conditioned medium and the PGC1α-conditioned one did not rescue the caFoxO3-reduced protein synthesis in myotubes (Figure 1d). However, only the PGC1α-conditioned one restrained the caFoxO3-induced proteolysis per hour by about 6% in myotubes (Figure 1f), indicating that some factors are released specifically in the medium of PGC1α-expressing cells, able to exert anti-catabolic but not pro-anabolic action on atrophying myotubes.

To further characterize these effects, we transfected myoblasts with a reporter plasmid where the gene for *Firefly Luciferase* is under the control of the MuRF1 promoter and concomitantly with a plasmid encoding *Renilla Luciferase* under the control of a constitutive promoter (HSV-thymidine kinase promoter) to normalize the data. We exposed these same myotubes 24 h later to 10 μM dexamethazone to induce atrophy in GFP or PGC1α-conditioned media for the next 24 h. As expected, dexamethazone induced MuRF1 (Figure 1g). Importantly, we found that the PGC1α-conditioned medium reduced the dexamethazone-induced expression of MuRF1 of myotubes in luciferase assays (Figure 1g), confirming through an unrelated approach the specific anti-catabolic action of the medium enriched with PGC1α-induced myokines.

### 2.2. Unlike PGC1β, Exogenous PGC1α Induces the Expression of Fibroblast Growth Factor 18 (FGF18), Natriuretic Peptide Precursor B (NppB), Amphiregulin (AREG) and Musclin in Myotubes and Tibialis Anterior (TA) Muscle

**Figure 2:**
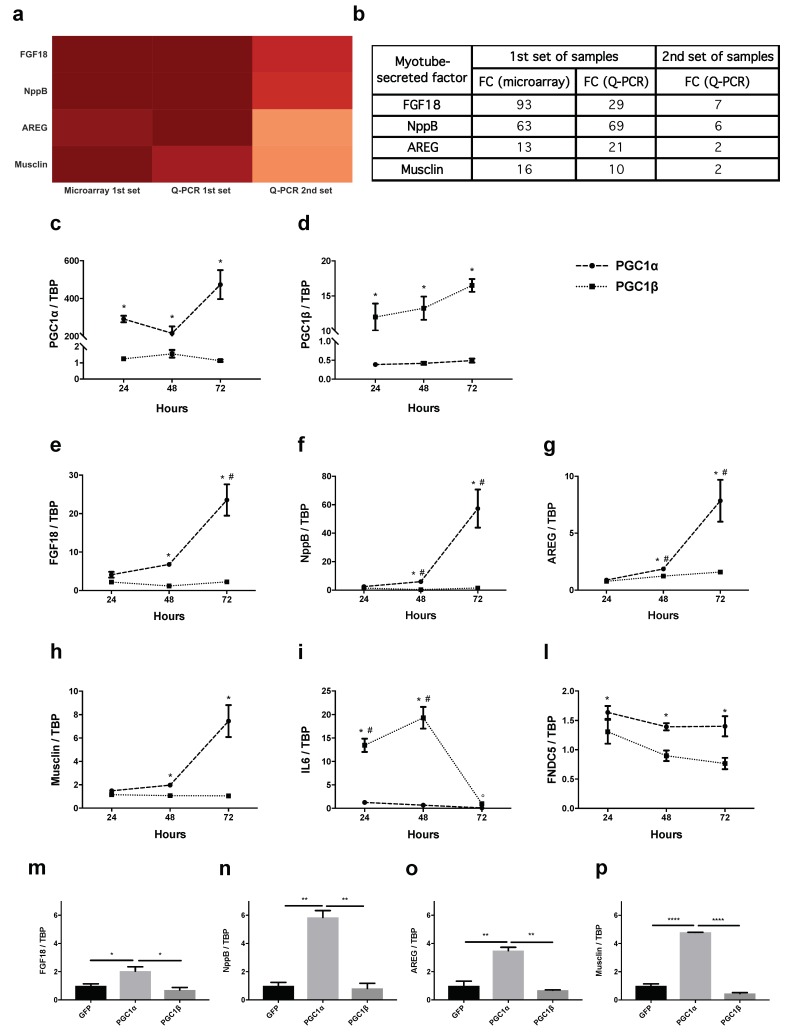
*Fibroblast Growth Factor 18 (FGF18), Natriuretic Peptide Precursor B (NppB), Amphiregulin (AREG)* and *musclin*, as well as *Fibronectin type III domain-containing protein 5 (FNDC5)*, are induced in myotubes and muscles expressing PGC1α but not PGC1β. Heat-map of the putatively secreted factors induced in 48 h-PGC1α-expressing myotubes with log2-fold change greater than 4, identified from the differential expression analysis and subsequent Gene Ontology (GO) enrichment (**a**). The table summarizes the fold induction of these factors in microarray and in quantitative polymerase chain reaction (Q-PCR) assays on the same samples as the microarray (1st set of samples) and on unrelated ones (2nd set of samples) (**b**). By Q-PCR, the expression of PGC1α (dashed line) and PGC1β (dotted line) in myotubes expressing PGC1α (**c**) or PGC1β (**d**) was monitored for the indicated times. Values are expressed as fold change over controls (GFP-expressing myotubes). The expression of *FGF18* (**e**), *NppB* (**f**), *AREG* (**g**), *musclin* (**h**) and, as further controls, *interleukin 6 (IL6)* (**i**) and *FNDC5* (**l**) was evaluated by Q-PCR in myotubes expressing PGC1α (dashed line) or PGC1β (dotted line) for 24, 48 and 72 h. Values are expressed as fold change over controls (GFP-expressing myotubes). *TATA binding protein (TBP)* was used as housekeeping gene. One-way ANOVA with repeated measures followed by Newman–Keuls post hoc test. * *p* ≤ 0.05 vs. controls (GFP-expressing myotubes), *n* = 3; # *p* ≤ 0.05 vs. 24 or 48 h (**e**) or vs. both other timepoints (**f**,**g**), *p* ≤ 0.05 vs. 24 or 48 h of PGC1α-related IL6 expression vs. controls (**i**). Tibialis Anterior (TA) of 10 week-old male BALB/c mice were electroporated for 14 days with plasmids for GFP or PGC1α or PGC1β. In vivo transfection was monitored with specific probes by Q-PCR (Figure S3a,b) and gene expression plotted as the fold change over controls (GFP-expressing muscles). *TBP* was used as housekeeping gene. The expression of *FGF18* (**m**), *NppB* (**n**), *AREG* (**o**) and *musclin* (**p**) are induced specifically by PGC1α, as well as *FNDC5* (Figure S3c). One-way ANOVA followed by Tukey’s test, * *p* ≤ 0.05, ** *p* ≤ 0.01, **** *p* ≤ 0.0001, *n* = 2–3.

To identify which factors induced by PGC1α exert such anti-catabolic action on atrophying myotubes, we screened out from the genes differently induced only in cells expressing PGC1α for 48 h, but not in uninfected or GFP-expressing ones, those encoding for proteins that potentially could be secreted extracellularly. To this end, we performed a directed Gene Ontology (GO) enrichment towards the “cellular component” annotation, selecting genes that belonged to the extracellular component (GO terms: “extracellular space” and “extracellular region”). We then selected potentially secreted factors specific to PGC1α-expressing cells, using the SwissProt database. We selected fibroblast growth factor 18 (FGF18), NppB, amphiregulin (AREG) and musclin (Figure 2a,b) because of their high degree of differential expression (at least log2-fold change >4) compared to GFP-expressing ones.

In quantitative polymerase chain reaction (Q-PCR) experiments, we validated these genes as induced by PGC1α on the same samples used for microarray-based assays (1st set of samples) and on other independent biological samples (2nd set of samples) (Figure 2a,b). In time-course experiments, myotubes were infected for 24, 48 and 72 h with adenoviruses expressing PGC1α (or PGC1β as further control, because it is an isoform of PGC1 that is not involved in muscle adaptation to aerobic exercise [34]). First, the infection was monitored over time with specific primers against PGC1 isoforms, showing increased gene expression of either *PGC1*α (Figure 2c) or *PGC1*β (Figure 2d) at all times. As expected, *FGF18* (Figure 2e), *NppB* (Figure 2f), *AREG* (Figure 2g) and *musclin* (Figure 2h) were induced 2–8 fold at 48 h and 8–60 fold at 72 h after adenoviral infection, confirming they were all modulated by PGC1α but not PGC1β.

As further controls, we measured the expression of *IL6*, which is induced in muscles by aerobic exercise [35] and that of *FNDC5*, which is the uncleaved precursor of irisin, a myokine whose induction and secretion is under PGC1α control [20]. *IL6* was induced between 13 and 20 fold by PGC1β 24 and 48 h from infection and decreased by PGC1α but only 72 h from infection (Figure 2i). As expected, *FNDC5* was induced at all times from PGC1α infection (but not after PGC1β) (Figure 2l), although much less than all the other genes that were PGC1α targets in our screening (Figure 2e–h). Results were similar in the Tibialis Anterior (TA) muscle of adult mice electroporated for 14 days with plasmids expressing PGC1α or PGC1β (Figure 2m–p and Figure S3a–c), extending in vivo the relevance of our findings in vitro.

### 2.3. Among the Newly Identified PGC1α-Related Myokines, only Musclin Is Strongly Reduced in TA and Plasma of C26-Bearing Mice

**Figure 3:**
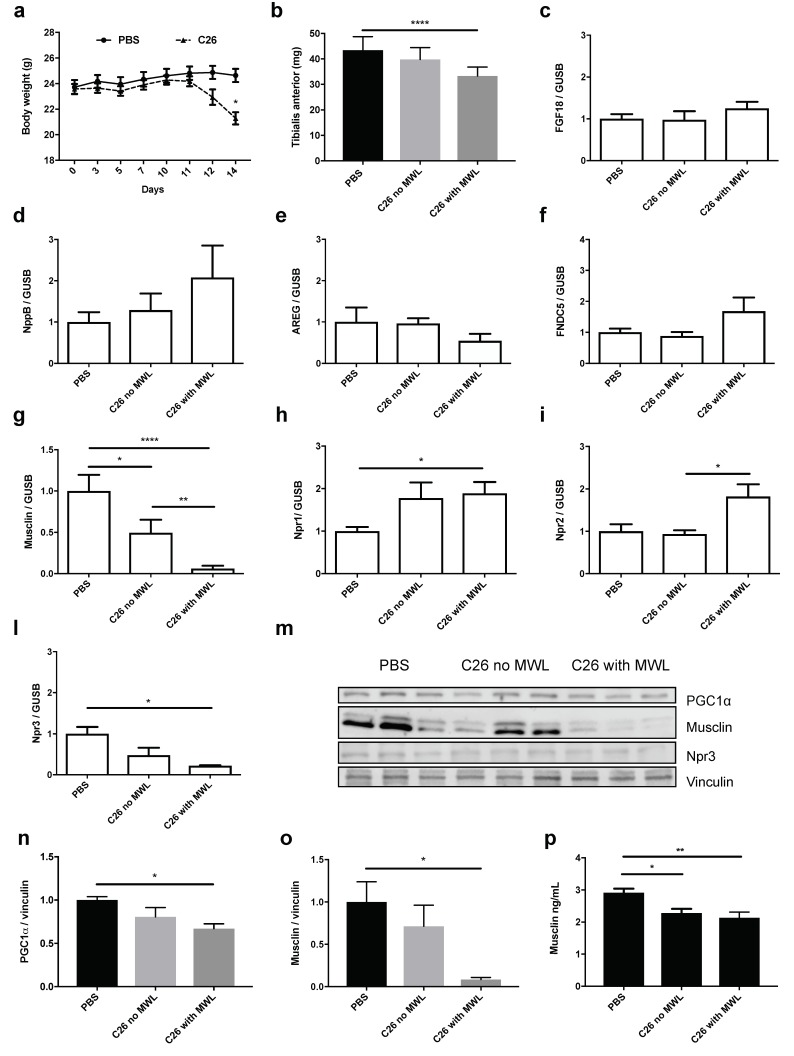
Only musclin is reduced in Tibialis Anterior (TA) and plasma of C26-bearing mice even at times when muscles and body weights have not yet been reduced. The body weights of the phosphate-buffered saline (PBS)-injected or C26-bearing mice, whose plasma has been tested in enzyme-linked immunosorbent assay (ELISA) to measure musclin, are shown in (**a**). One-way ANOVA with repeated measures followed by Newman–Keuls post hoc test. * *p* ≤ 0.05, *n* = 8–10. TA weights of PBS-injected or C26-bearing mice with or without muscle weight loss (MWL) are plotted in (**b**). One-way ANOVA followed by Tukey’s test, **** *p* ≤ 0.0001, *n* = 6–9. The fold change compared to PBS-injected mice of the expression of *FGF18* (**c**), *NppB* (**d**), *AREG* (**e**), *FNDC5* (**f**), *musclin* (**g**), *Npr1* (**h**), *Npr2* (**i**) and *Npr3* (**l**) was measured in TA of C26-bearing mice with or without MWL, in other words with or without atrophy of TA. β*-glucuronidase (GUSB)* was used as housekeeping gene. One-way ANOVA followed by Tukey’s test, * *p* ≤ 0.05, ** *p* ≤ 0.01, **** *p* ≤ 0.001, *n* = 5–16. The protein contents of PGC1α, musclin, Npr3 and vinculin, used as loading control, are shown in Western Blotting (WB) (**m**), with the related band quantitation for PGC1α (**n**) and musclin (**o**). Both were strongly reduced in cachectic TA muscle from C26-bearing mice with MWL. One-way ANOVA followed by Tukey’s test, * *p* ≤ 0.05, *n* = 3. The plasma concentration of musclin was already reduced in C26-bearing mice at times when their body weights were still comparable to PBS-injected mice (**p**). One-way ANOVA followed by Tukey’s test, * *p* ≤ 0.05, ** *p* ≤ 0.01, *n* = 7–12.

Since PGC1α is reduced in muscles of cancer-bearing rodents [16,36] including mice with colon adenocarcinoma (C26) [37], we asked whether the expression of the newly identified PGC1α-related myokines also changed in muscle in this condition. So, we measured the expression of the myokines in TA of C26-bearing mice at earlier times when muscle weight had not yet decreased (C26 without muscle weight loss: MWL, 10 days post-tumor injection) and later on, when TA were already reduced in size (with MWL, 14 days post-tumor injection) (Figure 3a,b). Interestingly, we found that the expression of *FGF18* (Figure 3c), *NppB* (Figure 3d), *AREG* (Figure 3e) and *FNDC5* (Figure 3f) was unaffected in these muscles. Conversely, the expression of *musclin* was already reduced in TA before wasting occurred and reached much lower levels in cachectic TA (Figure 3g). Similarly, unlike *IL6*, *FGF18* and *NppB*, only the expression of *musclin* tended to be lower in TA from nude mice bearing a rare human renal carcinoma, RXF393 (Figure S4) [38]

Given the high and prompt reduction of *musclin* expression in muscle during C26-induced cachexia, we measured the expression of its receptor *Npr3* and the related ones, *Npr1* and *2* by Q-PCR. While *Npr1* and *2* were induced in MWL conditions (Figure 3h,i), only *Npr3* was reduced in muscle with wasting (Figure 3l). Results were similar with Western Blotting (WB) (Figure 3m–o), where protein content for PGC1α, musclin and Npr3 was reduced in cachectic TA of C26-bearing mice, further confirming the correlation between PGC1α and musclin.

Even more importantly, in the plasma of C26-bearing mice at times when their body weights did not differ from that of PBS-injected ones and muscles were not yet wasted, the circulating levels of musclin had already decreased (Figure 3p), supporting it as a possible biomarker predicting cancer cachexia.

### 2.4. Mice Deficient of Musclin Undergo more Muscle Depletion during Growth of Two Unrelated Tumors: Lewis Lung Carcinoma (LLC) or Methylcholanthrene-Induced Sarcoma 101 (MCG101)

In order to understand if musclin may be necessary to contrast cancer-induced muscle wasting, we injected cachexia-inducing tumors in wild-type (WT) or musclin KO C57BL6/J mice. We could not inject C26 cells in these mice because these cells have been obtained from BALB/c mice and would have been rejected if implanted in a different strain.

**Figure 4:**
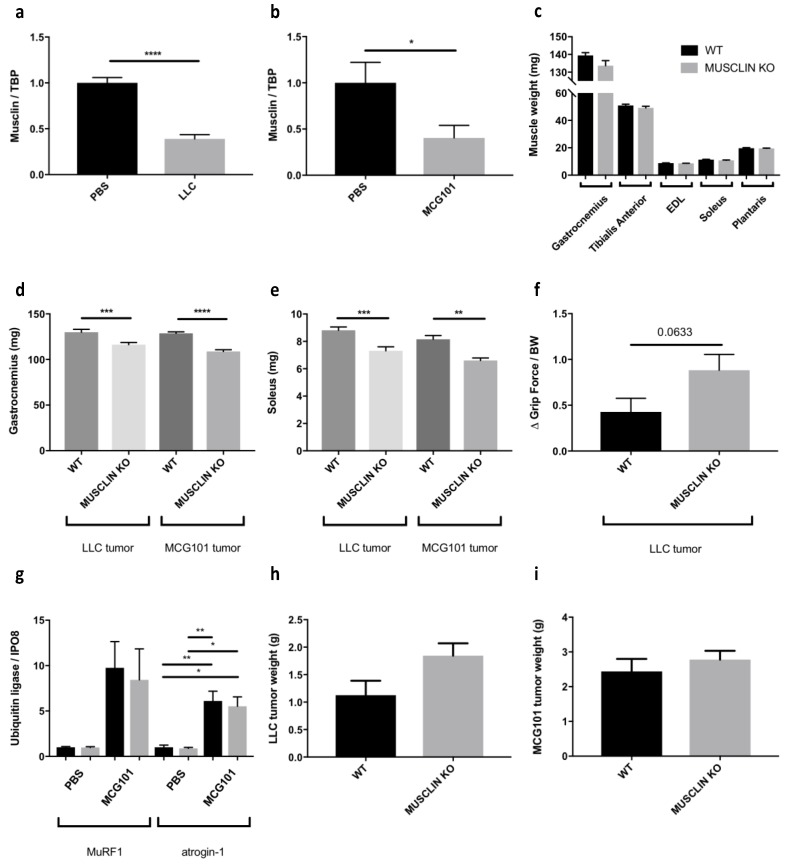
Musclin-knockout (KO) mice undergo more muscle depletion in response to cachexia-inducing tumors, as Lewis lung carcinoma (LLC) and methylcholanthrene-induced sarcoma 101 (MCG101). The expression of *musclin* has been measured in Tibialis Anterior (TA) from LLC- (**a**) or MCG101-bearing mice (**b**) at times when they suffer from cachexia. *TBP* was used as housekeeping gene and values normalized to phosphate-buffered saline (PBS)-injected mice. Unpaired t test for (**a**), **** *p* ≤ 0.0001, *n* = 8–9. Unpaired t test for (**b**), * *p* ≤ 0.05. The weights of gastrocnemii, TA, Extensor Digitorum Longus (EDL), solei and plantaris do not differ between age-matched wild-type (WT) and musclin-KO mice (**c**). Unpaired *t*-test, *n* = 6–10. Both gastrocnemii (**d**) and solei (**e**) are smaller in LLC or MCG101-bearing musclin-KO mice than WT counterparts. Unpaired *t*-test, ** *p* ≤ 0.01, *** *p* ≤ 0.001, **** *p* ≤ 0.0001, *n* = 14 for LLC *n* = 10–12 for MCG101. The grip strength between day 12 and day 27 of WT and musclin-KO mice injected at day 0 with LLC cells has been normalized for body weight and seems to be more reduced in musclin-KO mice (**f**). Unpaired t test, p value is shown, *n* = 8–9. The expression of *MuRF1* and *atrogin-1* is induced in gastrocnemii of mice injected with MCG101 cells for 14 days. In muscles from musclin-KO mice, this induction is not further exacerbated (**g**). *Importin 8* (*IPO8)* was used as a housekeeping gene and values normalized to PBS-injected mice. For color legend refer to Figure 4c. One-way ANOVA followed by Tukey’s test, * *p* ≤ 0.05, ** *p* ≤ 0.01, *n* = 4–6. Either LLC (**h**) or MCG101 cells (**i**) grow similarly when injected subcutaneously in mice, regardless the presence or not of musclin in the mouse genetic background. Unpaired *t*-test, *n* = 7 for (**h**); *n* = 5–6 for (**i**).

Lewis lung carcinoma (LLC) and methylcholanthrene-induced sarcoma 101 (MCG101) are tumors known to cause body weight loss with muscle depletion in C57BL6/J mice when implanted subcutaneously [39,40]. Consistently, we found that musclin was downregulated either in cachectic TA from LLC- (Figure 4a) or MCG101-carrying mice (Figure 4b), as we previously found in muscles from C26-bearing mice (Figure 3g). Notably, the sizes of several muscles (gastrocnemius, TA, Extensor Digitorum Longus or EDL, soleus and plantaris) did not differ in age-matched WT mice from musclin KO ones (Figure 4c), indicating that the solely absence of musclin is not sufficient to cause muscle wasting in tumor-free mice. Interestingly, both gastrocnemii and solei of musclin-KO mice injected with LLC or MCG101 cells displayed decreased weights when compared to cachectic WT mice (Figure 4d,e), suggesting that musclin loss exacerbates muscle wasting during cancer. Furthermore, we found that musclin KO mice tended to display reduced grip strength between days 12 and 27 from LLC cell injection than WT (Figure 4f). When we measured the mRNA expression of different markers of protein catabolism, such as *MuRF1* and *atrogin-1* among others, we did not find any difference in gastrocnemii of MCG101-bearing WT and musclin KO mice, suggesting that other unknown players may be affected by musclin depletion (Figure 4g). Finally, these tumors reached comparable sizes when injected in WT or musclin-KO mice, indicating that musclin does not grossly impact on tumor growth in vivo (Figure 4h,i). 

### 2.5. Exogenous Expression of Musclin or Its Receptor Npr3 Partially Preserves Fiber Areas during C26 Growth in Mice

**Figure 5:**
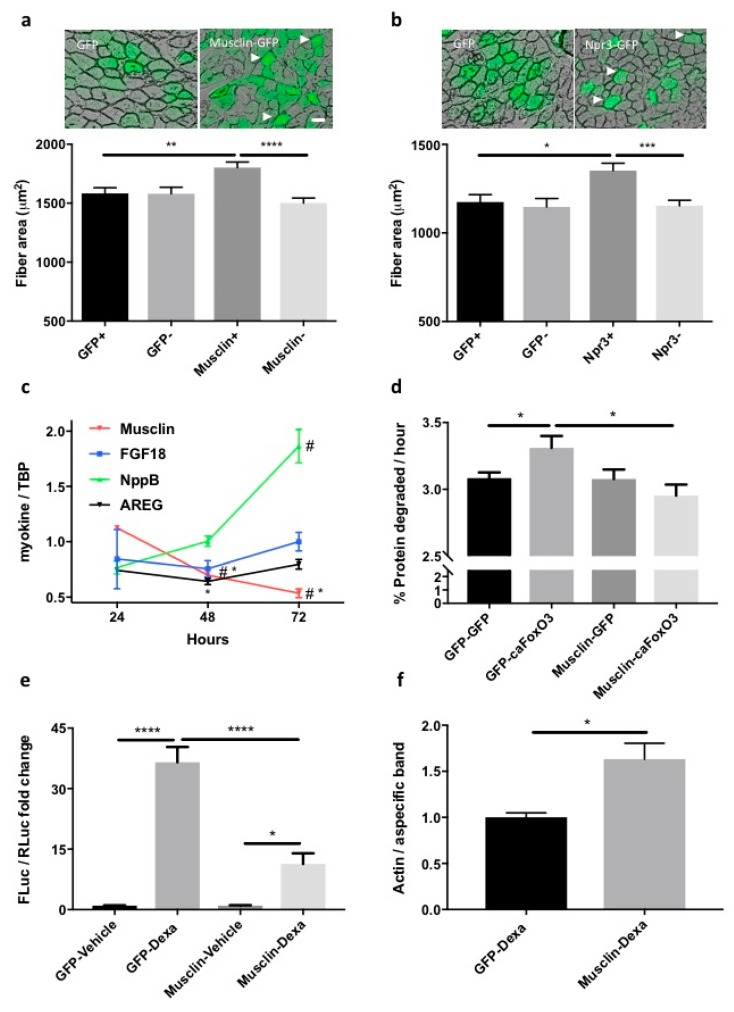
Overexpression of musclin (or its receptor) preserves the fiber area of Tibialis Anterior (TA) during C26 tumor growth in mice, reduces the proteolysis of long-lived proteins in atrophying myotubes, and restrains MuRF1-based signaling in dexamethasone-treated cells. A representative transverse section of fibers electroporated with musclin-GFP-carrying plasmids (**a**) or Npr3-GFP-expressing ones (**b**) from C26-bearing mice is shown. Scale bar, 50 μm. Arrowheads indicate enlarged fibers as example. The mean cross-sectional area (CSA) of fibers electroporated with the indicated plasmids is plotted for C26-bearing mice, indicating preserved fiber area in musclin or Npr3-expressing fibers (**a**,**b**). We analyzed 5–6 legs for a total of 102 GFP-expressing fibers and 102 GFP-negative ones, 140 musclin-positive and 140 musclin-negative fibers, 171 Npr3-positive and 171 Npr3-negative fibers. One-way ANOVA followed by Tukey’s test, * *p* ≤ 0.05, ** *p* ≤ 0.01, *** *p* ≤ 0.001, **** *p* ≤ 0.0001. Myotubes infected for 24, 48 and 72 h with adenoviruses for GFP of caFoxO3 and the expression of the indicated myokines is shown as the fold change over control. Only *musclin* expression is reduced by caFoxO3 overexpression. *TBP* was used as housekeeping gene (**c**). One-way ANOVA with repeated measures followed by Newman–Keuls post hoc test. * *p* ≤ 0.05 vs. controls (GFP-expressing myotubes); # *p* ≤ 0.05 vs. 24 h (*musclin*) or vs. both other timepoints (*NppB*), *n* = 3. Three-day differentiated myotubes were transfected with plasmids for GFP or musclin and the next day, these cells were further infected with adenoviruses for GFP or caFoxO3, as indicated. The caFoxO3-induced rates of degradation of long-lived proteins are lowered by co-expressed musclin (**d**). One-way ANOVA followed by Tukey’s test, * *p* ≤ 0.05, *n* = 5–6. The dexamethasone-induced MuRF1 promoter-FLuc signal is reduced in myoblasts expressing musclin or Npr3 but not in GFP-expressing ones. Myoblasts treated for 24 h with 10 μM dexamethasone had first been transfected with MuRF1 promoter-FLuc plasmid and TK promoter-RLuc plasmid in combination with the other plasmids. The results of one representative experiment repeated three times are shown (**e**). One-way ANOVA followed by Dunnett’s test, * *p* ≤ 0.05, **** *p* ≤ 0.0001, *n* = 3. Densitometric analysis shows that actin protein is spared in dexamethasone-treated myotubes expressing musclin with respect to GFP-expressing ones (**f**). A non-specific band of about 300 kDa found with actin antibody was used as loading control. Unpaired t test, * *p* ≤ 0.05, *n* = 3.

Oxidative myofibers that are rich in PGC1α are more resistant than glycolytic ones to tumor-induced atrophy [15], but it is not known yet whether this resistance relies on a specific fiber type-related myokine that acts in an autocrine way. We predicted that musclin might confer protection against cancer-induced atrophy for two main reasons. First, we found its endogenous expression strongly correlated with that of PGC1α in murine muscles with different metabolism (Extensor Digitorum Longus or EDL, solei) (Figure S3d) (r = 0.8822 and *p* = 0.0003). Second, in muscles electroporated with PGC1α-expressing vectors (Figure S3a), the fiber distribution of PGC1α co-localized with that of induced musclin (Figure 2p and Figure S3e). However, to test whether ectopic expression of musclin in muscles results in fiber preservation during C26 growth, we electroporated musclin-encoding plasmids in TA of C26-bearing mice and after 14 days, the mice were sacrificed and muscles analyzed, as we have previously done in [33]. Unlike GFP-expressing fibers that atrophy like their neighboring GFP-negative ones, fibers transfected in vivo for musclin had a bigger mean cross-sectional area than musclin-negative ones within the same muscle (Figure 5a), excluding paracrine effects for musclin for reasons we did not explore in depth. Similar results were obtained for Npr3-expressing plasmids (Figure 5b), indicating that in C26-related cachexia, neither musclin nor its receptor becomes rate-limiting in atrophying TA. Interestingly, in vivo musclin transfections in TA of C26-bearing mice also tended to reduce endogenous *MuRF1* (Figure S5).

To characterize more in depth the mechanism by which musclin exerts anti-atrophic effects, we used cultured myotubes where we can measure precisely any changes in protein homeostasis. In myotubes differentiated for various days, the expression of *Npr1* and *Npr3*, but not of *Npr2* (Figure S6a–c) decreased, at times when *ANP* and *musclin* expressions were mostly stable (Figure S6d,e). Consistent with the in vivo reduction of musclin in cachexia, myotubes expressing caFoxO3 for 24, 48 and 72 h display progressively reduced expression of *musclin*, unlike the other myokines that were mostly unchanged, with the exception of *NppB* mainly induced during atrophy (Figure 5c) and *FNDC5* reduced at 24 and 48 h (Figure S2d). These caFoxO3-expressing cells have higher rates of degradation of long-lived proteins, which is fully prevented by musclin-GFP but not GFP (Figure 5d), in agreement with data on supernatants from PGC1α-expressing myotubes (Figure 1g).

Finally, we found that musclin-GFP was able to restrain the dexamethazone-induced expression of MuRF1 of myoblasts in luciferase assays (Figure 5e), and to stabilize the protein content of long-lived proteins, such as actin, in dexamethasone-treated myotubes (Figure 5f).

### 2.6. Running Protects Gastrocnemius from C26-Induced Atrophy and Restores PGC1α, Musclin and Npr3 Expression in Mice

**Figure 6:**
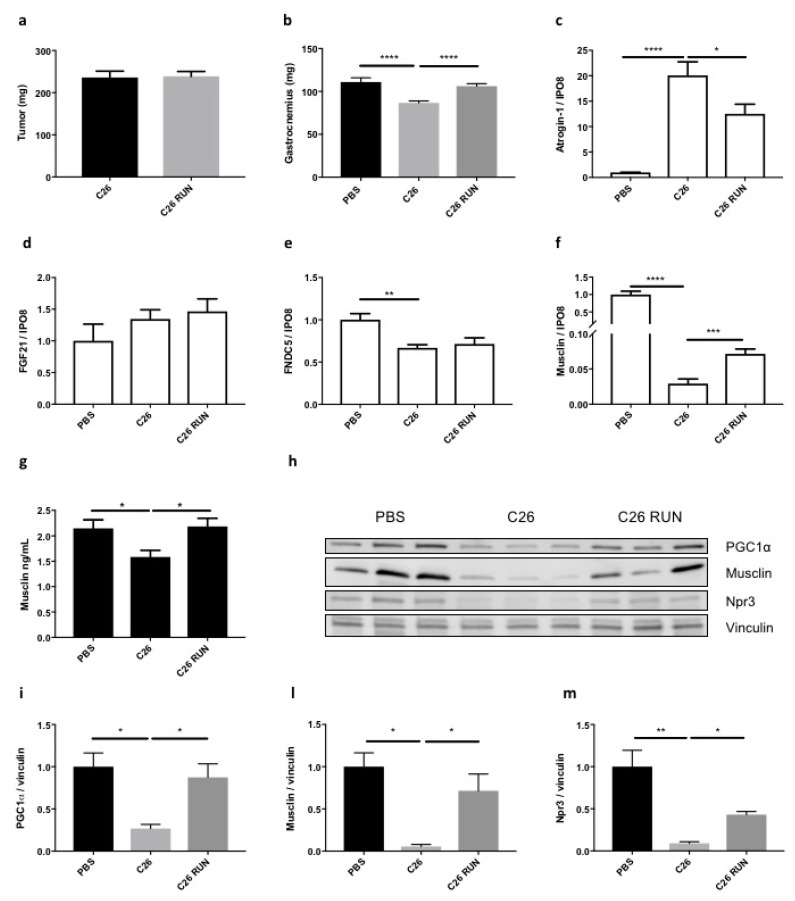
A five-day running schedule fully protects gastrocnemius from wasting in C26-bearing mice, reversing musclin reduction in muscle and plasma. C26 tumor size is not affected by five-day running in mice (**a**), *n* = 10. The gastrocnemius weight is fully preserved in C26-bearing mice in the running schedule (**b**). One-way ANOVA followed by Tukey’s test, **** *p* ≤ 0.0001, *n* = 4–9. C26-induced *atrogin-1* expression in gastrocnemius is restrained in the running-trained mice (**c**). While running has no effect on the expression of *FGF21* (**d**) or *FNDC5* (**e**), the expression of *musclin* in gastrocnemius (**f**) and the plasma concentration of musclin (**g**) are respectively partially and fully recovered in five day-trained mice. *IPO8* was used as housekeeping gene. One-way ANOVA followed by Tukey’s test, * *p* ≤ 0.05, ** *p* ≤ 0.01, *** *p* ≤ 0.001, **** *p* ≤ 0.0001 *n* = 8–10. In WB, the protein content of PGC1α, musclin and its receptor Npr3 in gastrocnemius of C26-bearing mice is restored to levels comparable to tumor-free mice after five-day running (**h**). Densitometric analysis is shown for PGC1α (**i**), musclin (**l**) and Npr3 (**m**). Vinculin served as loading control. One-way ANOVA followed by Tukey’s test, * *p* ≤ 0.05, ** *p* ≤ 0.01, *n* = 3.

We previously showed that musclin levels may be raised in muscles and plasma of mice running on treadmill for five days [24]. To learn whether running also increases musclin in cancer-bearing mice, we subjected to this training program mice that had been subcutaneously injected with C26 tumor 10 days earlier; we dissected and weighed their muscles three hours after the last running session. The total distance run in five days by these mice ranged between 2340 to 2700 meters, while PBS-injected mice ran 2700 meters, completing the exercise session, as expected (data not shown). This running protocol did not change the tumor size of mice at death (Figure 6a), but fully protected gastrocnemius weight from C26-induced cachexia (Figure 6b), restraining *atrogin-1* induction (Figure 6c). Gastrocnemii of trained C26-bearing mice showed no change in the expression neither of an AKT-related myokine, as *FGF21* (Figure 6d) nor of a PGC1α-induced myokine, such as *FNDC5* (Figure 6e). These same muscles when interrogated for proteins implied in AKT pathway indicated no AKT activation, because AKT, 4EBP-1, mTOR and S6K were no more phosphorylated upon running (Figure S7). This can be explained by “anabolic resistance”, typical of cachexia [41]. Only the expression of *musclin* was partially recovered in gastrocnemius from trained C26-bearing mice (Figure 6f).

Even more surprisingly, the plasma concentration of musclin increased by reaching control levels in C26-bearing mice subjected to running training (Figure 6g), mirroring what happens in muscle weight recovery. Notably, the C26-induced plasma IL6 was partially restrained in trained mice (Figure S8). Both musclin and its receptor Npr3 were strikingly restored to normal protein levels in gastrocnemii of trained C26-bearing mice as well as PGC1α (Figure 6h–m), indicating that the PGC1α/musclin/Npr3 axis can still be stimulated by running in cachectic mice.

### 2.7. Musclin Expression Does Not Change in Myristoylated AKT-Expressing Myotubes or in Hypertrophied Plantaris

Muscle adaptation to anaerobic exercise mainly consists of AKT activation with subsequent increased protein synthesis and hypertrophy [42]. Transgenic mice conditionally expressing AKT1 in skeletal muscles display impressive muscle hypertrophy [43].

**Figure 7:**
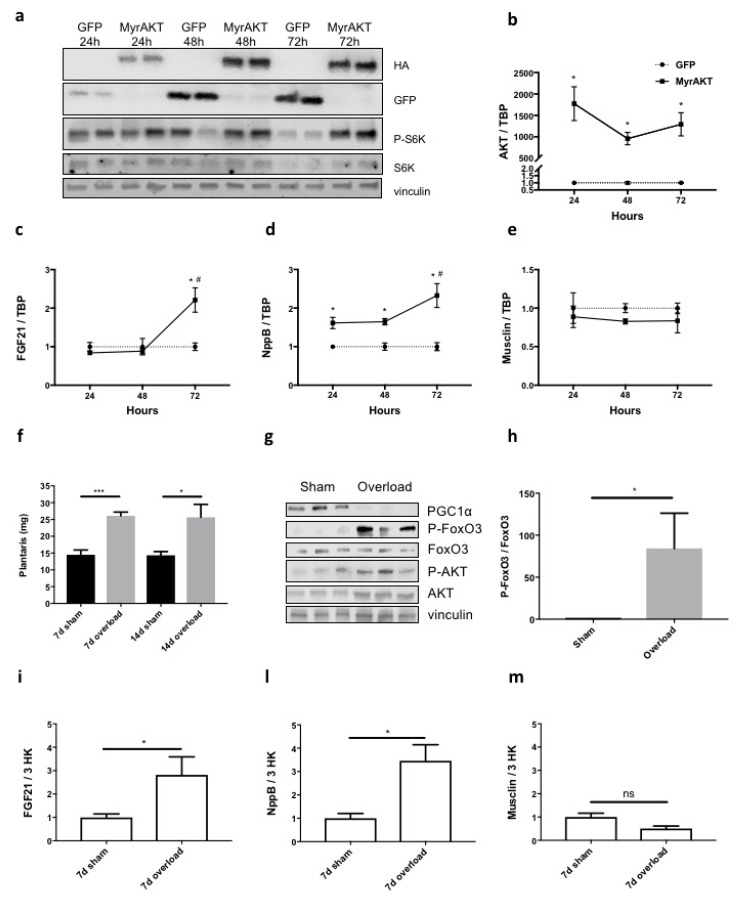
Unlike *FGF21* and *NppB*, *musclin* expression does not change in myotubes expressing myristoylated AKT (MyrAKT) for various times or in hypertrophied plantaris. By WB, we monitored the protein expression of MyrAKT-HA in myotubes infected for 24, 48 and 72 h with antibodies against hemagglutinin (HA) tag. As expected, one of its main representative substrates, S6K, was more phosphorylated over time, specifically in MyrAKT-expressing myotubes but not in GFP-expressing ones. Vinculin was used as loading control (**a**). The expression of *AKT* (**b**), *FGF21* (**c**), *NppB* (**d**) and *musclin* (**e**) was measured in myotubes expressing GFP (controls) and MyrAKT for the times indicated. *TBP* was used as housekeeping gene. One-way ANOVA with repeated measures followed by Newman–Keuls post hoc test, * *p* ≤ 0.05 vs. controls (GFP-expressing myotubes); # *p* ≤ 0.05 vs. 48 h (**d**) or vs. both other timepoints (**c**), *n* = 3. The weight of plantaris after surgical removal of its synergist muscles increased after 7 or 14 days compared to contralateral sham-operated plantaris in 10-week-old male BALB/c mice (**f**). Ratio paired *t*-test, * *p* ≤ 0.05, *** *p* ≤ 0.001, *n* = 4–5. The protein content of PGC1α, FoxO3, p-FoxO3 (Ser253), AKT and p-AKT (Ser473) is blotted for lysates of plantaris from three mice 7 days after their synergist muscles were surgically removed from the right leg (**g**). Vinculin served as loading control. The densitometric analysis shows that the ratio of p-FoxO3/FoxO3 normalized for vinculin, strongly increased in the plantaris overloaded for 7 days (**h**). Ratio paired *t*-test, * *p* ≤ 0.05, *n* = 3. The expression of *FGF21* (**i**), *NppB* (**l**) and *musclin* (**m**) was measured in plantaris hypertrophied or not for 7 days. Three housekeeping genes (*IPO8*, *GUSB*, *TBP*) served to normalize the data. Only *musclin* expression did not change in hypertrophied plantaris. Ratio paired *t*-test, * *p* ≤ 0.05, *n* = 4–5.

To understand whether the newly identified PGC1α-related myokines were specifically induced by PGC1α (that partly recapitulates some aspects of aerobic exercise) (Figure 1b) and not by a constitutively active form of AKT (myristoylated AKT: MyrAKT) (that partially recapitulates instead some effects of anaerobic exercise), we measured gene expression of proteins of interest in myotubes expressing MyrAKT for 24, 48 or 72 h. We monitored the content of the protein (Figure 7a) and mRNA (Figure 7b) for AKT over time by WB and Q-PCR, respectively, and found it highly expressed at all times. Subsequently, the phosphorylation of one of the main substrates of AKT, p70S6K increased 48 and 72 h post-infection (Figure 7a). The expression of AKT for 48 h resulted in increased total RNA content by about 40% (Figure S1a). All these parameters are indicative of increased protein synthesis, suggesting that MyrAKT-expressing myotubes somehow undergo hypertrophy that is a typical hallmark of strength exercise. Moreover, *FGF21*, an AKT-induced myokine [21] was raised in MyrAKT-expressing myotubes (Figure 7c), further validating our assays. Notably, while the expression of *NppB* was induced by MyrAKT in myotubes (Figure 7d), that of *musclin* was unaffected (Figure 7e). 

To rule out any aspecific role of musclin in muscle size control, in mice we unilaterally ablated the synergist muscles of plantaris (soleus and gastrocnemius) to cause hypertrophy of the plantaris. This overloaded muscle, being characterized by AKT activation, recapitulates at least one of the main muscle adaptations to anaerobic exercise [44,45]. Either 7 or 14 days from surgery, overloaded plantaris almost doubled in weight with respect to the contralateral one (Figure 7f). When we compared protein lysates of the plantaris overloaded for seven days to their normal counterparts, AKT was found activated and, as a result, one of its main targets, FoxO3 was more phosphorylated (i.e., inactivated) (Figure 7g,h). Strikingly, the overall protein content of PGC1α diminished in the overloaded plantaris (Figure 7g). Finally, seven days overloading caused transient induction of *FGF21* (Figure 7i) or *NppB* (Figure 7l) in plantaris at times when *musclin* expression remained mostly unchanged or even tended to lower levels (Figure 7m), suggesting its preferential involvement more in aerobic- than anaerobic-like muscle adaptations.

## 3. Discussion

Cachexia is a major unsolved medical problem during cancer treatment, with adverse effects on the patients prognosis. Physical activity is recommended for cancer patients to obviate cachexia [46], but the molecular mechanisms of the beneficial effects of exercise against cancer-induced muscle wasting are mainly unknown. To our knowledge, the present study is the first to compare in mice the expression of myokines induced by different types of exercise to identify those with therapeutic potential against muscle atrophy during cancer (for a review on this topic, see [19]).

We initially used PGC1α-infected myotubes because this reproduced to a certain extent some aspects of the adaptation to aerobic exercise (induced *MRPS33*, enhanced oxidative phosphorylation). We also employed MyrAKT-infected myotubes where some of the effects of anaerobic exercise are recapitulated through phosphorylation of substrates that enhance protein synthesis at the ribosome (ribosomal protein S6 kinase β 1, S6K), leading to increased overall RNA content [42]. PGC1α-infected myotubes served for the identification of specific myokines that we validated in Tibialis Anterior muscle electroporated with PGC1α, then in running mice (i.e., aerobic exercise) and in mice subjected to compensatory hypertrophy of the plantaris (to somehow obtain muscle growth as after anaerobic exercise). According to our data, five day-running sessions caused PGC1α induction in gastrocnemius muscles of cancer-bearing mice with prior increased phosphorylated AMPK in muscles (data not shown). Hypertrophied plantaris increased AKT-mediated phosphorylation of FoxO3, indicative of AKT activation [44] and even caused a surprising attenuation of PGC1α protein, perhaps because of preferential growth of fibers less rich in mitochondria (glycolytic fibers), as shown in the same model but in rats [47]. As further controls, we measured specific myokines induced by PGC1α (FNDC5, precursor of irisin) [20] or by AKT (FGF21) [21], in both models throughout the study: these were always affected, as expected, both in vitro and in vivo, further supporting our findings. 

We found *FGF18*, *musclin*, *NppB and AREG* as muscle-secreted factors induced by PGC1α in myotubes and adult muscles. The PGC1α-related induction of *AREG*, a member of the epidermal growth factor family, is consistent with data showing induction of *AREG* in remobilized soleus after 10 days of hindlimb immobilization in young rats [48]. Intriguingly, only musclin was specifically induced by PGC1α and running, but not by MyrAKT or in hypertrophied plantaris, indicating it to be engaged more in aerobic-, than anaerobic exercise-like muscle adaptations. This contrasts with previous findings [24,25,49], showing that musclin production is triggered by AKT activation/FoxO1 inhibition, when exposed to short bouts of exercise, transient Ca_2_^+^ ionophore application, or fasting/refeeding. These conditions associated with well-established short-living AKT activation, in contrast to persistent muscle overload or expression of constitutively active AKT models, which we used here. As such, these different dynamics of AKT activation [42,50,51,52,53] could result in the observed discrepancies of the downstream signaling and explain the differences from our results, where musclin was not induced by MyrAKT in vitro in four day-differentiated immortalized C2C12 myotubes at any time tested (24, 48 and 72 h post-infection). However, the induction of musclin by PGC1α we observed agrees with data from Staiger et al., who found musclin induced in myotubes by a peroxisome proliferator-activated receptor γ (PPARγ) agonist but not by a PPARα one [54] and PPARγ is known to enable PGC1α to interact with multiple transcription factors [55]. Consistent with our data, serum from 29- to 34-month-old transgenic mice overexpressing PGC1α in their muscles have increased musclin than their age-matched WT counterparts [56]. PGC1α is unable to bind directly the DNA, but promotes gene transcription through binding with various transcription factors. CCAAT/enhancer binding protein β (i.e., C/EBPβ) is a transcription factor able to bind PGC1α [57] and very recently it has been reported by chromatin immunoprecipitations that C/EBPβ binds the promoter of musclin and promotes its expression [58]. For the above reasons, we believe it could be a good candidate to test in future studies. 

We also previously reported musclin as induced by uphill running in mice [24]. Our present study shows that restoring musclin in cachectic muscles either through a genetic approach or uphill running is beneficial to counteract atrophy during cancer cachexia. This agrees with reports showing that especially aerobic activity can alleviate cancer cachexia [22,59], also because type I fibers are more resistant to atrophy than type II [15], while AKT overactivation was even found in cachectic muscles [23]. Interestingly, the drop in plasma concentration of musclin was fully recovered when C26-bearing mice were subjected to five days of uphill running sessions at moderate velocity, a condition that seems not to imply resistance exercise as others did [60], because *FGF21* was not increased at mRNA level in these trained muscles. Notably, a well-known PGC1α-related myokine, *FNDC5* (irisin) being downregulated in cachectic gastrocnemius (but not in TA)—although much less than musclin—is not reversed in gastrocnemius from treadmilled C26 mice, possibly excluding it among the mediators of the anti-atrophic effects of exercise in this model.

Musclin is greatly reduced in skeletal muscle of transgenic mice overexpressing the transcription factor FoxO1 [49], that agrees with its steep downregulation we observed in muscle of C26 mice [61] or more slightly in immunodeficient mice bearing human kidney cancer RXF393. Even if musclin was the least induced of all the other PGC1α-related myokines in our microarrays, it has the unique feature of being strongly reduced in atrophying caFoxO3-expressing myotubes, in both cachectic TA and PGC1α-losing gastrocnemii from C26-bearing mice and in TA from mice bearing LLC or MCG101 tumors. Infused musclin was found to support physical performance in mice [24]. Along the same line, Ishikawa et al. very recently showed increased expression of musclin in vitro during myotube differentiation or transcription factor C/EBP homologous protein (i.e., CHOP)-mediated endoplasmic reticulum stress [62], which is also involved in exercise tolerance in mice [63]. Since muscle strength is already reduced in C26-bearing mice before body wasting occurs [64], and we found a trend towards reduced grip strength of LLC-bearing musclin KO mice with respect to WT, we believe that reduction in musclin may account for the increased propensity to fatigue of cancer-bearing mice at earlier times. 

There is an urgent need to identify early circulating markers of cancer cachexia, because detecting muscle atrophy in advance in these patients by monitoring muscle mass is impeded by the wide variability of muscle mass in the general population and by the high cost and low accessibility of the instrumentation to measure it (computerized tomography-scan, dual-energy X-ray absorptiometry or DEXA, bioimpedance) [64]. Some markers of cancer cachexia have been identified (growth differentiation factor 15, i.e., GDF-15 or parathyroid hormone release peptide, i.e., PTHrP) but none of them have been shown to change before the cachexia appears [6], so that “omics” studies are in development to identify novel muscular or circulating markers of cachexia [65]. Reduced levels of circulating musclin from plasma anticipates cachexia in cancer-bearing mice that have not yet shown signs of body or muscle wasting, supporting musclin as an early biomarker of cancer cachexia. At that time, cancer-bearing mice eat just like controls, so the drop in circulating musclin cannot be explained by reduced food intake, as others reported in muscles from fasted mice [25], but we did not observe it in plasma of mice fasted for 16 or 48 h (data not shown). Since musclin is mainly expressed by skeletal muscles [25] but also by bones [26], its presence in plasma may reflect mainly its production by muscles that are much more vascularized than bones and more represented than bones in the entire body in humans as in mice. Musclin measurements in blood may be useful to identify in advance patients at risk of developing cachexia, who can be advised to increase their daytime aerobic exercise or to adhere to other anti-cachexia suggestions. 

In electroporation experiments, the size of the electroporated fibers expressing exogenous musclin, but not of the adjacent non-electroporated ones, was preserved, thus excluding any paracrine action of secreted musclin, contrarily to what we had previously shown for stromal cell-derived factor 1 (i.e., SDF1 or CXCL12) [33]. Musclin has sequence homologies with the atrial natriuretic peptides (ANP, BNP, CNP), with which it competes for binding to Npr3 [28] and it probably has a short plasma half-life, as the other members of the family have (ANP, 2 min, BNP, 20 min, CNP, 2.6 min) [27]. As a consequence, musclin must be constantly introduced at local/systemic levels to ensure a continuous beneficial effect, by restraining the caFoxO3-enhanced proteolysis typical of atrophy, as we showed at least in vitro. Furthermore, musclin has two cutting sites for serine proteases [25], probably responsible for a mitigated protective effect in the muscle of C26 cachectic mice.

Since similar results were obtained by overexpressing Npr3 in vivo, we believe that neither musclin nor its receptor, even if strongly downregulated in cachectic muscle, become rate-limiting, supporting this pathway as still likely to be responsive to potentially related future drugs. This is consistent with our data obtained in musclin-KO mice, where the total absence of ubiquitous musclin further exacerbates muscle depletion and, to a lesser extent, muscle force loss during cancer. Since Npr3 seems not to have an enzimatic cytosolic domain, it may work as a scavenger receptor, removing from the extracellular environment active ligands of Npr1 and Npr2, such as ANP, BNP and CNP [27]. Thus, musclin may potentiate NPs signaling through their cognate receptors by binding and subsequently removing Npr3 from the cell surface. Increased cyclic guanosine 3′,5′-monophosphate (i.e., cGMP) production following stimulation by natriuretic peptides in muscles, as in myocytes, results in increased oxidative metabolism of muscle, in other words, more lipid utilization and mitochondria biogenesis [66,67]. 

Oxidative fibers are more resistant to cancer-induced wasting, so we believe that musclin downregulation in plasma and muscle during cachexia may attenuate NP-based signaling, contributing to reduced oxidative metabolism (i.e., PGC1α downregulation) typical of cancer-induced muscle wasting among others. So, NP-driven myofiber-type shift could be beneficial during cachexia and exogenous musclin, but not Npr3, electroporation may promote it. Our data showing at later times increased expression of both Npr1 and 2 in cachectic TA of C26-carrying mice may suggest a possible compensatory activation of Npr1/2 axis during C26-induced cachexia. Nonetheless, since either electroporated musclin or Npr3 protein content in muscle spared muscle mass, we suspect that the beneficial effects of (musclin or) Npr3 act through the ability of this activated receptor to couple with heterotrimeric Gαi2 proteins [68] that in turn are involved in muscle hypertrophy [69]. Even more interestingly, it seems that NPs–Npr axes, especially cardiac-derived ANP and muscle-restricted Npr1, are induced by aerobic exercise in humans [66] and participates to muscle adaptation to aerobic training. Nonetheless, a better understanding of the role of NP-based signalling in skeletal muscle is needed to design possible drugs with anti-cachexia action in this tissue.

## 4. Materials and Methods 

### 4.1. Cell Culture

C2C12 (ATCC, Manassas, VA, USA), a myoblast cell line from the C3H mouse strain, was grown in DMEM (Dulbecco’s Modified Eagle’s Medium, Gibco, Waltham, MA, USA), supplemented with fetal bovine serum (FBS) (Euroclone, Pero, Italy) and 2 mM L-glutamine, and maintained in culture at 37 °C with 5% CO_2_. Myoblasts differentiate into myotubes when they reached 80% confluence and were cultured for four days in DMEM, 2 mM L-glutamine (BioWest, Nuaillè, France), supplemented with horse serum (HS) (Euroclone, Pero, Italy), at 37 °C and 8% CO_2_. The differentiation medium was changed every two days. C26 is a colorectal adenocarcinoma cell line from BALB/c mice, grown in DMEM supplemented with 10% FBS and 2 mM L-glutamine at 37 °C with 5% CO_2_. These cells were a kind gift from Prof. Mario Paolo Colombo (IRCCS-Istituto Nazionale dei Tumori, Milan, Italy) and authenticated as in [70]. The cells used were not contaminated by mycoplasma. LLC cells derive from C57BL6/J mice and are grown in DMEM supplemented with 10% FBS and 2 mM L-glutamine at 37 °C with 5% CO_2_. MCG101 is a sarcoma cell line from C57BL6/J mice, grown in McCoy’s 5A medium supplemented with 10% FBS and 2 mM L-glutamine at 37 °C with 5% CO_2_. MCG101 cells were kindly donated by Prof. Anders Blomqvist (Linköping University, Sweden), while LLC cells were a kind gift from Prof. Paola Costelli (University of Turin, Italy). 

### 4.2. Adenoviruses and Plasmids

Myotubes were infected on the fourth day of differentiation with adenoviruses encoding for GFP as control, PGC1α and GFP separated by internal ribosome entry site (IRES) sequence, PGC1β and GFP separated by IRES sequence, caFoxO3 or MyrAKT-HA. Myotubes were infected in six-well plates with 1.5 mL/well of differentiation medium and 0.25 μL/well of adenoviral preparations for GFP, PGC1α, PGC1β and MyrAKT, or 1 μL/well for caFoxO3, based on preliminary experiments to determine the smallest amount of non-toxic virus needed to infect all myotubes. The next day, cells were washed four times with 2 mL PBS/well and incubated with differentiation medium for another 24 h. Adenoviruses were kindly donated by Prof. A.L. Goldberg (Harvard Medical School, Boston, USA), while that encoding for MyrAKT was a generous gift from Prof. Ken Walsh (Boston University). pCMX-GFP plasmid was a kind gift from Prof. Kakizuka, Japan. Plasmids for pIRES-GFP, PGC1α-IRES-GFP and PGC1β-IRES-GFP were kindly donated by Prof. Jeff Brault (East Carolina University, NC). Plasmid pcDNA3 for MyrAKT1-HA was kindly provided by Prof. Ken Walsh (Boston University). Vectors for murine musclin-GFP and Npr3-GFP were obtained from Origene. For the experiments with supernatants, myotubes were infected on the fourth day of differentiation in six-well plates. The next day, cells were washed four times with 2 mL PBS/well and incubated with differentiation medium for another 48 h, when the conditioned medium was centrifuged at 1200 rpm for 5 min and the pellet-free supernatant used to treat myotubes.

### 4.3. Microarrays

We used SurePrint G3 Mouse Gene Expression Microarray Kit v2 8x60K (design 028 005 ID, Agilent Technologies, Santa Clara, CA, USA) for microarray analysis of myotubes expressing adenovirus for PGC1α for 48 h and, as controls, non-infected cells or myotubes infected with adenovirus for GFP for 48 h. RNA labeling and hybridization were carried out according to the manufacturer’s protocols. After hybridization, arrays were washed to remove unspecific hybridization and scanned with a laser confocal scanner (G2565B, Agilent Technologies, Santa Clara, CA, USA), according to the manufacturer’s directions. Agilent Feature Extraction (AFE), version 11, was used to quantify the fluorescent signals, using default analysis parameters.

Raw data were pre-processed, removing features marked as unreliable by the scanning software in at least 60% of the samples, and then normalized using the “quantile” method. Differential analysis was carried out with linear models for microarray analysis [71], correcting the resulting *p*-values for multiple testing with the false discovery rate (FDR) method [72]. When we compared GFP-infected vs. not infected myotubes in microarray analyses, we performed a differential expression analysis at high (FDR < 0.01) and medium (FDR < 0.05) stringency, and no gene passing the significance threshold cut-offs was identified. Thus, we chose to compare the data to GFP-infected myotubes, namely controls. Only genes with a corrected p-value less than 0.05 and regulated at least two-fold compared to controls were considered significant. In accordance with the Minimal Information About a Microarray Experiment (MIAME; http://fged.org/projects/miame/) guidelines, raw and processed data have been submitted to the Array Express repository (ID pending).

Functional analysis was carried out on the Kyoto Encyclopedia of Genes and Genomes (i.e., KEGG) database [73] using Fisher’s Exact Test [74] and the resulting *p*-values were corrected with the FDR method. Gene Ontology data analysis was done using the topGO R package (https://www.bioconductor.org) [75], to calculate non-redundant enrichment on cellular component annotations.

### 4.4. Protein Degradation and Synthesis in C2C12 Myotubes

C2C12 myotubes expressing GFP or caFoxO3-GFP for at least 16 h were incubated with 3H-tyrosine (2 μCi/mL; PerkinElmer, Waltham, MA, USA) for 24 h to label long-lived proteins and then processed as in [76]. In these experiments, treatments with the media conditioned by PGC1α- or GFP-expressing cells for 72 h were added simultaneously with the 3H-tyrosine and kept during washes. Total protein synthesis was calculated by incorporation of 3H-tyrosine into cell proteins, following the procedure described in [33].

### 4.5. Luciferase-Based Assays

Luciferase assay was done on C2C12 myoblasts grown in a 24-well plate and transfected with GFP or GFP-musclin-expressing plasmids in combination with murine MuRF1 promoter-FLuc plasmid, kindly supplied by Dr. Nicoletta Rizzi (University of Milan, Italy) and pRL-TK plasmid (Promega, Madison, WI, USA) using Lipofectamine 2000 (Life Technologies Europe BV, Carlsbad, CA, USA), according to the manufacturer’s directions. The next day they were treated with vehicle or 10 μM dexamethasone for 24 h with or without supernatants from GFP or PGC1α expressing myotubes. Firefly and Renilla luciferase activities were measured in cell lysates using the Dual-Luciferase Reporter Assay System (Promega, Madison, WI, USA); the signal was quantitated using a luminometer (Glomax 20/20 single tube luminometer, Promega, Madison, WI, USA).

### 4.6. Mice and Tumor Model

C26 cells were seeded at a density of 17000 cells/cm^2^ and 48 h later injected subcutaneously (1 × 10^6^ cells) into the upper right flank of 8 week-old male mice (BW 22–24g) (Harlan Laboratories, Lesmo, Italy). Mice were weighed the day of the injection, then every two days until they began to lose weight, after that they were monitored daily. In electroporation studies in tumor-free mice, animals were killed 7 or 14 days after in vivo muscle transfection. Mice (at least five/group) were randomly allocated to different groups based on body weights and sacrificed when they showed clear signs of distress or 14 days after tumor transplant, when they were electroporated. Body weights and tumor growths were monitored as detailed in [33]. LLC cells were seeded at a density of 25,000 cells/cm^2^ and 48 h later injected subcutaneously (1 × 10^6^ cells) into the upper right flank of 8-week-old male mice (body weight (BW) 22–24g) (Harlan Laboratories, Lesmo, Italy). MCG101 cells were seeded at a density of 12,000 cells/cm^2^ and 48 h later injected subcutaneously (0.5 × 10^6^ cells) into the upper right flank of 8-week-old male mice (BW 22–24 g) (Harlan Laboratories, Lesmo, Italy).

Mice were trained on an open treadmill (TSE-System, Homburg, Germany) for 20 min/day for three days, at 3.5m/min, with 15° uphill inclination (acclimation time) and then run for five consecutive days for 45 min/day at 12 m/min, with 15° uphill inclination (training time), strictly following the protocol described in [24]. Muscles were isolated 3 h after the last running session.

Procedures involving animals and their care were conducted in conformity with institutional guidelines in compliance with national and international laws and policies. The Mario Negri Institute for Pharmacological Research IRCCS adheres to the principles set out in the following laws, regulations, and policies governing the care and use of laboratory animals: Italian Governing Law (D.lgs 26/2014; Authorisation n° 19/2008—A issued 6 March 2008 by Ministry of Health); Mario Negri Institutional Regulations and Policies providing internal authorisation for persons conducting animal experiments (Quality Management System Certificate—UNI EN ISO 9001:2015—Reg. n° 6121); the National Institutes of Health (NIH) Guide for the Care and Use of Laboratory Animals (2011 edition) and European Union (EU) directives and guidelines (European Economic Community (EEC) Council Directive 2010/63/UE). The Statement of Compliance (Assurance) with the Public Health Service (PHS) Policy on Human Care and Use of Laboratory Animals has been recently reviewed and will expire in February 2020 (Animal Welfare Assurance #167/2017PR). All animal protocols conform to the Guide for the Care and Use of Laboratory Animals generated by the Institute for Laboratory Animal Research, National Research Council of the National Academies, and are approved by the University of Iowa Institutional Animal Care and Use Committee.

### 4.7. Musclin (Ostn)-Knockout (KO) Mouse Model

Mice were created as previously described [24]. In short, vector construction and targeted KO strategy were designed together with genOway, where mice were generated on C57BL/6 genetic background through deletion of a 2.1-kb sequence flanking Ostn exon 2, resulting in inactivation of the ATG and signal peptide. Forelimb grip strength was determined using a grip strength meter, equipped with a triangular pull bar (Columbus Instruments INC, Columbus, OH, USA). Each mouse was subjected to 5 consecutive tests to obtain the average peak value, which was normalized to body weight, as done in [77]. The measurements were taken on days 12 and 27 after tumor cell injections.

### 4.8. RNA Isolation from Cultured Cells or Muscles and Reverse Transcription

Total RNA was isolated from cells or muscles with QIAzol Lysis Reagent (Qiagen, Hilden, Germany) and miRNeasy Kit (Qiagen, Hilden, Germany). RNA concentration, purity and integrity were measured in a spectrophotometer (NANODROP 1000, ThermoFisher Scientific, Waltham, MA, USA). Cells from one well of a six-well plate were resuspended in 700 μL of QIAzol and the frozen muscle of interest was cut perpendicular to the tendon and supplemented with 700 μL of QIAzol Lysis Reagent (Qiagen, Hilden, Germany). It was then lysed with T25 digital Ultra-Turrax homogenizer (IKA). From this point onwards, the extraction procedure was identical to that for RNA extraction from cells. A High-Capacity cDNA Reverse Transcription Kit (Applied Biosystems, Waltham, MA, USA) was used for the reverse transcription of RNA to cDNA. Transcription was done for 1 µg of RNA in 40 μL, according to the following reaction schedule: 25 °C for 10 min; 37 °C for 2 h; 85 °C for 5 min. Samples were then stored at −20 °C.

### 4.9. Quantitative Real-Time Polymerase Chain Reaction (PCR)

Analysis of mRNA/µg in muscle was done using TaqMan reverse transcription reagents (Life Technologies Waltham, MA, USA) or the fluorescent intercalating DNA SYBR Green (Qiagen, Hilden, Germany). *GUSB* (β-Glucuronidase) or *TBP* (Tata binding protein) or *IPO8* (Importin 8) were used as housekeeping genes. Genes analyzed in this study and their related sequences are listed in Supplementary Table S1. To normalize for three housekeeping genes, we used the ratio between the arithmetic average and the geometric average of the ng of cDNA of each gene, as described in [78]. We loaded each well of 96-well plates with 20 ng of cDNA (2 μL) obtained with retro-transcription, supplemented with either the primers and the SYBR Green (Qiagen, Hilden, Germany) mix or the probe and the TaqMan Mix (ThermoFisher Scientific, Waltham, MA, USA). In both cases, water was added to a volume of 11 μL. The PCR cycle for Real-Time PCR was as follows: step 1, 95 °C for 15 min; step 2, 95 °C for 25 sec; step 3, 60 °C for 1 min; repeating steps 2 and 3 for 40 cycles. The instrument used for these assays is a 7900HT Fast Real-Time PCR System (ThermoFisher Scientific, Waltham, MA, USA).

### 4.10. Protein Extraction and Western Blot

Total proteins were extracted from myotubes using lysis buffer (1% TritonX-100, 10 mM Tris pH 7.6, 50 mM NaCl, 30 mM sodium pyrophosphate, 50 mM NaF, 5 mM ethylenediaminetetraacetic acid or EDTA, Na_3_VO_4_ 0.1 mM), with the final addition of 4% sodium dodecyl sulphate (SDS) and protease inhibitors (Roche, Basel, Switzerland). The protein lysates of TA or plantaris or gastrocnemius muscles (20 slices 20 µm thick) were prepared using a buffer (1% Triton X-100, 50 mM Tris pH 7.5, 150 mM NaCl, 10 mM MgCl_2_, 0.5 mM dithiothreitol or DTT, 1 mM EDTA, 10% glycerol) with the final addition of 2% SDS and protease inhibitors (Roche, Basel, Switzerland). The final protein concentration was determined by bicinchoninic acid or BCA (Pierce, Waltham, MA, USA) or Bradford (Biorad, Hercules, CA, USA) assays. Then, 10–40 µg of proteins were added to Laemmli sample buffer (Biorad, Hercules, CA, USA) previously mixed with 10% β-mercaptoethanol (Sigma, St. Louis, MO, USA), and boiled at 97 °C for 7 min. 

Proteins were separated by electrophoresis on 4%–20% sodium dodecyl sulphate-polyacrylamide gel electrophoresis (SDS-PAGE) (BioRad) and transferred to a polyvinylidene difluoride membrane (GE Healthcare, Chicago, IL, USA) that was then saturated for 2h at room temperature in a solution of 5% bovine serum albumin (BSA) or milk in buffer that is 20 mM Tris, 150 mM NaCl and 0.1% Tween-20 (Sigma, St. Louis, MO, USA) (TBS-T buffer). The membrane was then incubated with the primary antibody O/N at 4 °C. The following primary antibodies were used: anti-musclin 1:200 custom rabbit immunoglobulin G (IgG) against 80–112 aminoacids of musclin peptide, 1:1000 anti-HA (BK2367, Cell Signaling, Danvers, MA, USA), 1:10,000 anti-GFP (A11122, Life Technologies Waltham, MA, USA), 1:1000 anti-P70-S6K (9202, Cell Signaling, Danvers, MA, USA), 1:1000 anti-phospho-T389S6K (9205, Cell Signaling, Danvers, MA, USA), 1:5000 anti-vinculin (V9264, Sigma, St. Louis, MO, USA), 1:1000 anti-Npr3 (GTX64458, Genetex, Irvine, CA, USA), 1:1000 anti-FoxO3 (2497, Cell Signaling, Danvers, MA, USA), 1:1000 anti-phospho-Ser253FoxO3 (9466, Cell Signaling, Danvers, MA, USA), 1:1000 anti-PGC1α (ab54481, Abcam, Cambridge, UK), 1:1000 anti-AKT (BK9272s, Cell Signaling, Danvers, MA, USA), 1:1000 anti phospho-Ser473AKT (BK9271S, Cell Signaling, Danvers, MA, USA), 1:1000 anti-atrogin-1 (ab168372, Abcam, Cambridge, UK), 1:5000 anti-actin (clone C4, MAB1501, Merck Millipore, Burlington, MA, USA). After O/N incubation with the primary antibody, the membrane was washed for 30 min with TBS-T and incubated for 1 h at room temperature with the secondary antibody, diluted in a solution of 1% BSA or milk in TBS-T. The membranes were then washed for 30 min in TBS-T to remove the excess of unbound antibody. Secondary antibodies were conjugated to alkaline phosphatase (Promega, Madison, WI, USA) and detected with CDP-star substrate (ThermoFisher Scientific, Waltham, MA, USA). Band intensities were analysed using ImageJ software (National Institutes of Health, Bethesda, MA, USA). 

### 4.11. Enzyme-Linked Immunosorbent Assay (ELISA) 

Musclin levels in murine plasma were measured in an enzyme-linked immunosorbent assay (ELISA). Plasma was collected in 0.5M EDTA as an anticoagulant, centrifuged 10 min at 10,000 rpm at 4 °C, and then stored at −80 °C. Musclin was quantified with a CUSABIO ELISA kit (Tema Ricerca S.r.l., Castenaso, Italy). Diluted plasma samples and standards were applied to the ELISA plates, and incubated for 2 h at 37 °C. Plates were then incubated with biotinylated antibody for 1 h at 37 °C. After three washes, plates were incubated with horseradish peroxidase (HRP)-avidin 1 h at 37 °C, then washed and incubated with the tetramethylbenzidine substrate for 15 min at 37 °C. Optical density was detected at 540 nm. Samples were loaded in duplicates. The detection range of the kit is 15.6–1000 pg/mL and the sensitivity is 3.9 pg/mL.

### 4.12. Electroporation of the TA with Plasmid DNA

The Endotoxin-Free Maxi Prep kit (Invitrogen, Carlsbad, CA, USA) was used to purify plasmids for muscle electroporation. The TA muscle was electroporated, with the animals anesthetized by inhalation of 3% isoflurane and 1% O_2_. Their legs were shaved and a linear incision of about 1 cm was made in the skin to expose the muscle. Using Dumont forceps with curved ends to lift the muscle, one electrode was placed under the TA, and 20 µg of plasmid DNA in 30 µL of 0.9% NaCl solution in H_2_O were injected with a 30-gauge Hamilton syringe. The other electrode was rolled over the muscle, which was electroporated with five pulses (21 V) of 20 millisec each, with a 200 millisec interval. Electroporation was done with the BTX ECM 830 Square Wave Electroporation System (Harvard Apparatus, Cambridge, MA, USA). Finally, the wound was sutured and disinfected with betadine. After 7–14 days, muscles were dissected and weighed by an individual unaware of the electroporation conditions.

### 4.13. Compensatory Hypertrophy

To induce compensatory hypertrophy of the plantaris, seven 10 week-old male BALB/c mice (Envigo, Lesmo, Italy) were subjected to unilateral removal of the synergist muscles. Briefly, the gastrocnemius and the soleus were ablated from the right leg, with the mice anesthetized by inhalation of 3% isoflurane and 1% O_2_. The left leg was sham operated. A linear incision was made in the ankle skin to expose the tendons. After isolation of the tendons of interest with Dumont curved forceps, they were cut off. The wound was sutured with surgical glue. To measure the plantaris hypertrophy, mice were sacrificed 7 or 14 days after surgery, when muscles were dissected and weighed.

### 4.14. Muscle Sample Processing and Fiber Size Measurements

Ten-μm thick cryosections of electroporated muscles were fixed in 4% paraformaldehyde. Using ImageJ software. CSA of transfected and untransfected fibers from the same muscle were measured in blind conditions. Pictures of myotubes and muscle fibers were acquired with an Olympus Microscope IX71 (20× magnification, 10× ocular lens, Olympus, Shinjuku, Japan) with Cell F (2.6 Build1210, Olympus, Shinjuku, Japan) imaging software for Life Science microscopy © (Olympus Soft Imaging solution GmbH, Munster, Germany). 

### 4.15. Statistical Analysis

Sample size was determined by power analysis with G*Power on the basis of similar experiments previously published by our laboratory. All the experiments were repeated at least twice. For statistical analysis, data (means ± standard errors of the mean or SEMs) were analyzed with GraphPad Prism 3.5 for Windows (Graph-Pad Software, San Diego, CA, USA) and Statview Software for Windows (SAS StatView for Windows Redmond, WA, USA), with the following statistical tests: ordinary one-way analysis of variance (ANOVA) for multiple comparisons followed by Dunnett’s or Newman–Keuls post hoc test, one-way ANOVA for repeated measures followed by Newman–Keuls test, unpaired t-test for comparisons of two groups. Ratio paired *t*-test was used for data from experiments on compensatory hypertrophy of the plantaris. *p*-values less than 0.05 were considered significant. 

## 5. Conclusions

Physical activity is important for preventing certain types of cancer [79] and to counteract cachexia in cancer patients, leading to better chances of recovery and/or response to therapy. Myokines, such as fibroblast growth factor 21 (i.e., FGF21), are released into the circulation in humans through physical activity and may have beneficial effects [80]. The molecular mechanisms of physical exercise-induced benefits are not fully understood, both in preserving health and slowing pathologies [81]. However, we believe we have identified a myokine regulated by PGC1α and/or aerobic exercise, with possible therapeutic action, that could hopefully be confirmed in cancer patients who are not able to do regular physical activity.

## Supplementary Materials

The following are available online at https://www.mdpi.com/2072-6694/11/10/1541/s1: Table S1. A list of all the genes with their related identification codes, and all the primers (and related vendors) used are reported. Figure S1. MyrAKT- or PGC1α-expressing myotubes recapitulate partly some features of anaerobic and aerobic exercise-like muscle adaptations, respectively. Figure S2. *Atrogin-1* and *MuRF1* are induced in myotubes expressing caFoxO3 for 24, 48 and 72 h, while *FNDC5* is reduced at 24 and 48 h. Figure S3. The expression of musclin correlates with that of endogenous PGC1α in various murine muscles and colocalizes with electroporated PGC1α in Tibialis Anterior (TA). Figure S4. Unlike the other PGC1α-related myokines, only the expression of *musclin* tends to be decreased in TA from RXF393-bearing mice. Figure S5. *MuRF1* transcript levels tend to be attenuated in TA from C26-bearing mice expressing musclin-GFP plasmids. Figure S6. In differentiating myotubes, the expression of *Npr1* and *3* decreases and that of *Npr2* increases at times when ligands of these receptors, *ANP* or *musclin*, are mostly stable. Figure S7. Five-day running does not induce AKT signaling in TA of C26-bearing mice. Figure S8. The plasma concentration of IL6 is increased in C26-bearing mice and partially restrained in five day-trained mice.
