# Supplementary material for: Musclin, A Myokine Induced by Aerobic Exercise, Retards Muscle Atrophy During Cancer Cachexia in Mice
Source: Cancers (Basel). 2019 Oct 12;11(10):1541. doi: 10.3390/cancers11101541 (PMC6826436; doi:10.3390/cancers11101541)
Supplement: Supplementary file 1 [file cancers-11-01541-s001.pdf]

Supplementary Material and Methods

Supplementary Table S1. A list of all the genes with their related identification codes, and all the primers (and related vendors) used are reported.

Gene symbol	***Company***	***Gene annotation***	***Assay name***	***Gene description***	***Forward sequence***	***Reverse Sequence***
MuRF1	Life Technologies	NM_001039048	Mm01185221	Muscle RING-finger protein-1	not available	not available
Atrogin-1	Life Technologies	NM_026346	Mm00499523	Muscle-specific F-box protein	not available	not available
FNDC5	Metabion	NM_027402		Fibronectin type III domain-containing protein 5	5' CAA CGA GCC CAA TAA CAA CA 3'	5' AGA AGG TCC TCT CGC ATT CTC 3'
FGF21	Metabion	NM_020013		Fibroblast growth factor 21	5' GCT GCT GGA GGA CGG TTA CA 3'	5' CAC AGG TCC CCA GGA TGT TG 3'
PGC1α	Metabion	NM_008904		Peroxisome proliferator-activated receptor γ co-activator 1α	5' TCA AGC CAA ACC AAC AAC TTT ATC T 3'	5' GGT TCG CTC AAT AGT CTT GTT CTC A 3'
GUSB	Metabion	NM_010368		β-Glucuronidase	5' TCG TAC CAG CCA CTA TCC CTA 3'	5' AAA ACT CTG AGG TAG CAC AAT GC 3'
TBP	Metabion	NM_013684		TATA binding protein	5' ACC CCA CAA CTC TTC CAT TCT 3'	5' TTT GAA GCT GCG GTA CAA TTC 3'
IPO8	Life Technologies	NM_001081113	Mm01255158	Importin 8	not available	not available
Musclin	Metabion	NM_198112		Musclin	5' GCA TCA CAG GAG TTT GGA ACA 3'	5' CAG ATC ATC AAG ACG CAG GA 3'
FoxO3	Metabion	NM_019740		Forkhead Box O3	5' TAC GAG TGG ATG GTG CGC TG 3'	5' AGG TTG TGC CGG ATG GAG TTC 3'
AKT	Metabion	NM_001165894		Phosphatidylinositol-3-kinase	5' TCA CCT CTG AGA CCG ACA CC 3'	5' ACT GGC TGA GTA GGA GAA CTG G 3'
PGC1β	Metabion	NM_001127330		Peroxisome proliferator-activated receptor γ co-activator 1β	5' TCC TGT AAA AGC CCG GAG TAT 3'	5' TAC TGG GTG GGC TCT GGT AG 3'
FGF18	Metabion	NM_008005		Fibroblast growth factor 18	5' GTG CTT CCA GGT TCA GGT GT 3'	5' AGC TGC TTC CGA CTC ACA TC 3'
Npr1	Life Technologies	NM_008727	Mm00435309	Atrionatriuretic peptide receptor 1 (A)	not available	not available
Npr2	Life Technologies	NM_173788	Mm00612889	Atrionatriuretic peptide receptor 2 (B)	not available	not available
Npr3	Life Technologies	NM_001039181	Mm00435329	Atrionatriuretic peptide receptor 3 (C)	not available	not available
AREG	Metabion	NM_009704		Amphiregulin	5' ACA GCG AGG ATG ACA AGG A 3'	5' CAA AGG TGC ACT GTG ATA ACG 3'
NppB	Metabion	NM_008726		Natriuretic peptide precursor B	5' GGT CCA GCA GAG ACC TCA AA 3'	5' CAG TGC GTT ACA GCC CAA A 3'
IL6	Metabion	NM_031168		Interleukin 6	5' TCT CTG CAA GAG ACT TCC ATC C 3'	5' TGA AGT CTC CTC TCC GGA CTT 3'
MRPS33	Life Technologies	NM_001010930	Mm03009791	Mitochondrial ribosomal protein S33	not available	not available
CMV	Metabion	NM_001157416		Cytomegalovirus	5' GGT CTA TAT AAG CAG AGC TG 3'	5' GTG GTA TGG CTG ATT ATG ATC AG 3'
ANP	Metabion	NM_008725		Atrial natriuretic peptide	5' TCG TCT TGG CCT TTT GGC T 3'	5' TCC AGG TGG TCT AGC AGG TTC T 3'

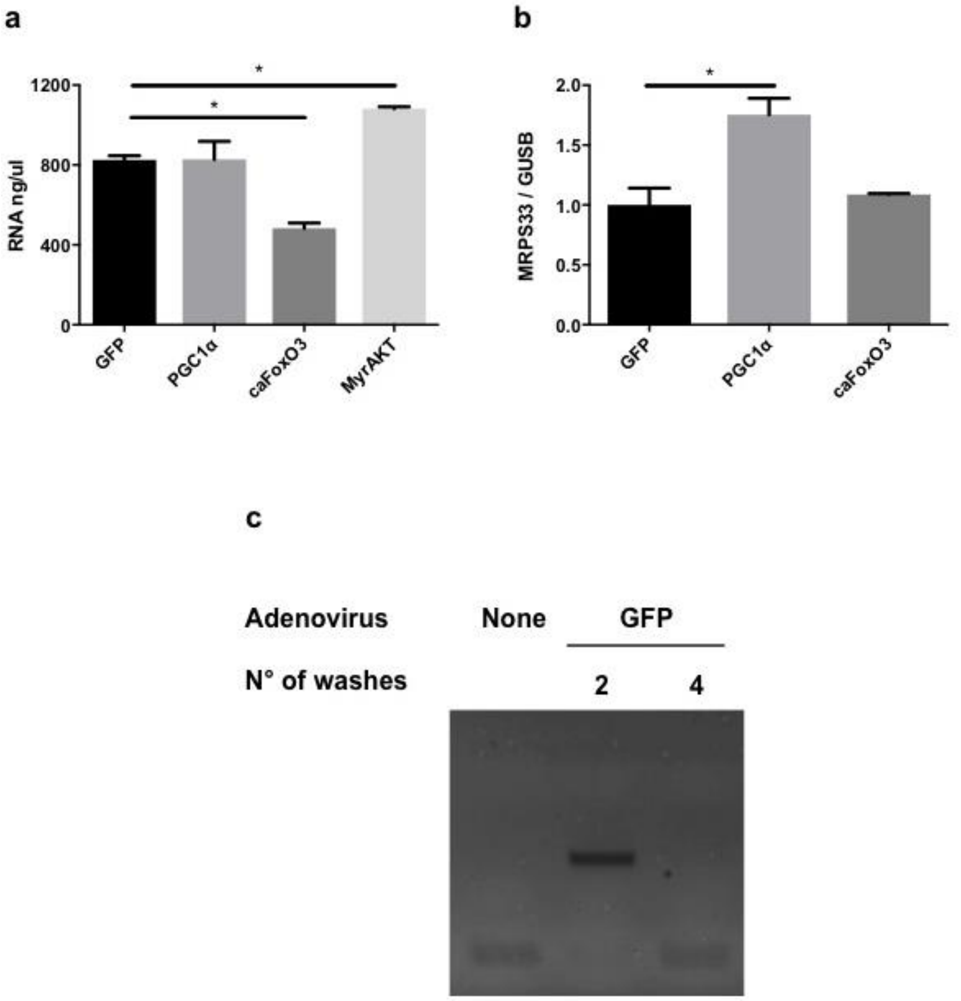

Figure S1. MyrAKT- or PGC1α-expressing myotubes recapitulate partly some features of anaerobic and aerobic exercise-like muscle adaptations, respectively. Four-day differentiated myotubes were infected for 48 h with adenoviruses encoding for GFP, PGC1α, MyrAKT or caFoxO3. Total RNA content decreased in caFoxO3-expressing myotubes and increased in MyrAKT-expressing ones (**a**). One-way ANOVA followed by Tukey's test, * $p \leq 0.05$, $n = 2$. The expression of a representative mitochondrial gene (*MRPS33*) increased only in PGC1α-expressing myotubes, as expected (**b**). *GUSB* was used as housekeeping gene. One-way ANOVA followed by Tukey's test, * $p \leq 0.05$, $n = 2$. Four, not two washes with PBS are needed to remove completely any adenoviral particle after overnight incubation with viruses-enriched medium (**c**). The supernatant from the three conditions was analyzed by PCR for the CMV-related sequence, present only in the adenovirus for GFP and not in cultured cells. The band in the gel where PCR products were loaded still shows the presence of adenovirus when only two washes were done after fresh medium replacement.

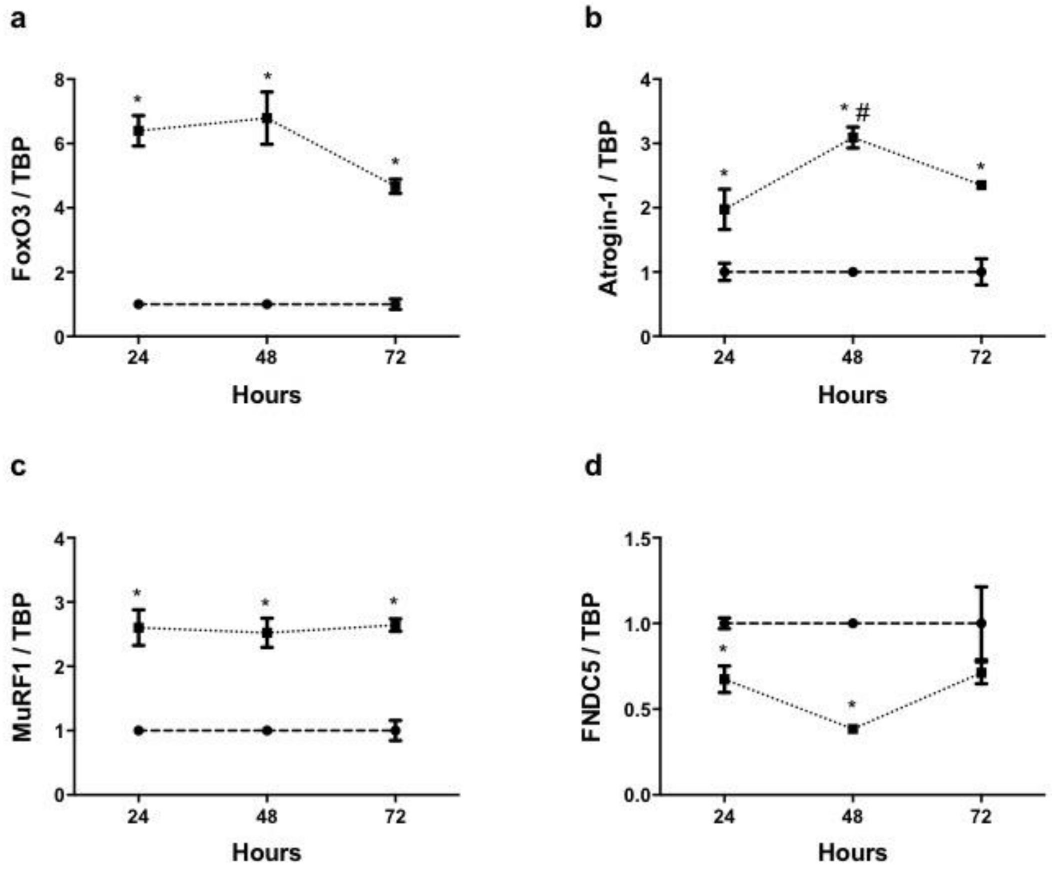

Figure S2. *Atrogin-1* and *MuRF1* are induced in myotubes expressing caFoxO3 for 24, 48 and 72 h, while FNDC5 is reduced at 24 and 48 h. By Q-PCR we monitored the expression of FoxO3 (dotted line) as the fold change from GFP-expressing cells (dashed line) in myotubes expressing caFoxO3-GFP (**a**) for the times indicated. The expression of *atrogin-1* (**b**) and *MuRF1* (**c**) increased, while that of *FNDC5* (**d**) decreased in caFoxO3-overexpressing myotubes. *TBP* was used as housekeeping gene. One-way ANOVA with repeated measures followed by Newman–Keuls post hoc test, * $p \leq 0.05$ vs. controls (GFP-expressing myotubes), $n = 3$; # $p \leq 0.05$ vs. 24 or 72 h (b).

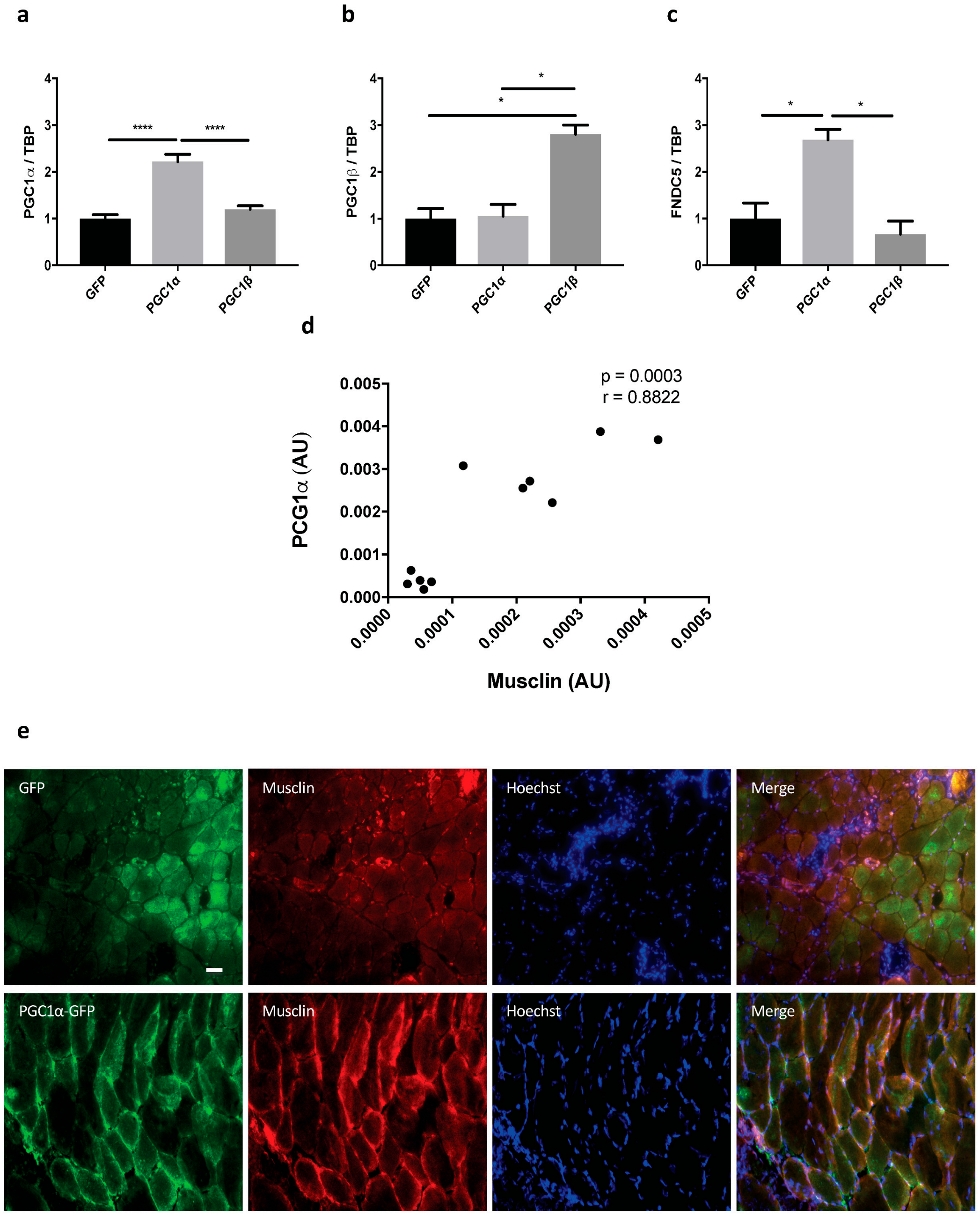

Figure S3. The expression of musclin correlates with that of endogenous PGC1α in various murine muscles and colocalizes with electroporated PGC1α in Tibialis Anterior (TA). TA of 10 week-old male BALB/c mice were electroporated for 14 days with plasmids for GFP or PGC1α or PGC1β. In vivo transfection was monitored with specific probes by Q-PCR (**a**,**b**) and gene expression plotted as the fold change over controls (GFP-expressing muscles). *TBP* was used as housekeeping gene. The expression of *FNDC5* is induced specifically by PGC1α, as expected (**c**). One-way ANOVA followed by Tukey's test, * $p \leq 0.05$, **** $p \leq 0.0001$, $n = 2$–3. Solei and EDL from BALB/c male mice were analyzed by Q-PCR to measure the expression of musclin and PGC1α (**d**). AU, Arbitrary units. Pearson's test is shown for correlation analysis. $p = 0.0003$ $n = 11$. TA of BALB/c male mice were electroporated with GFP-expressing or PGC1α-encoding plasmids. After 14 days, they were sacrificed and TA cut transversally and stained for endogenous musclin and nuclei with HOECHST. There is a high degree of colocalization between musclin and PGC1α. Scale bar, 25 µm (**e**).

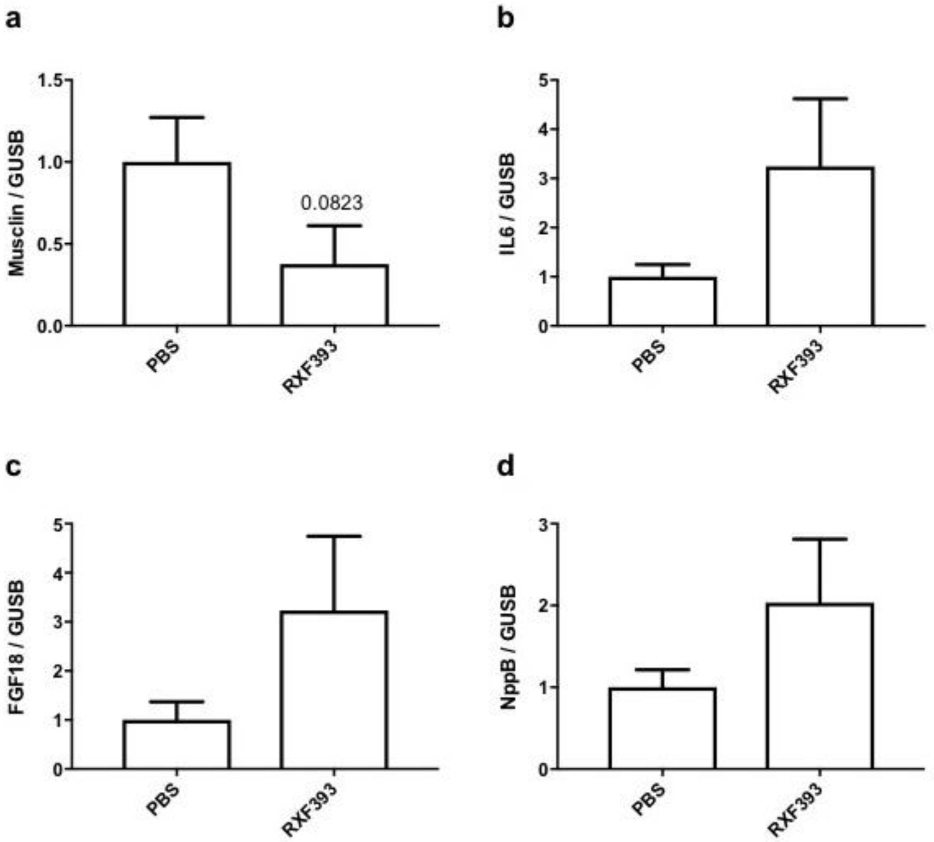

Figure S4. Unlike the other PGC1α-related myokines, only the expression of *musclin* tends to be decreased in TA from RXF393-bearing mice. The gene expression of *musclin* (**a**), *IL6* (**b**), *FGF18* (**c**) and *NppB* (**d**) was measured in Q-PCR in TA from PBS-injected and RXF393-bearing mice. *GUSB* was used as housekeeping gene. Unpaired t-test gave the p value reported for musclin, *n* = 6–7.

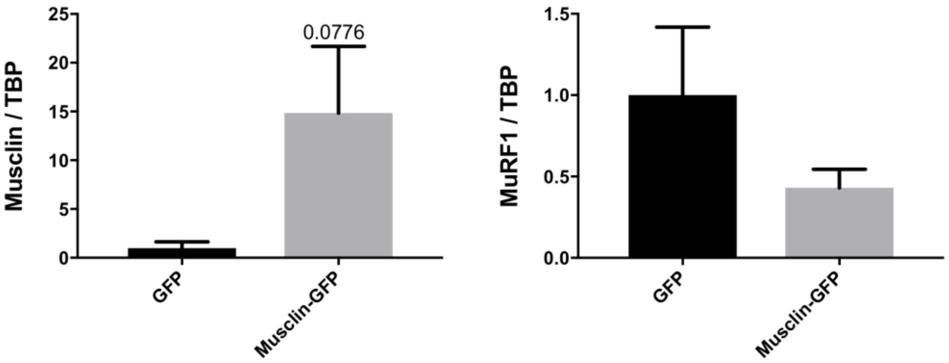

Figure S5. *MuRF1* transcript levels tend to be attenuated in TA from C26-bearing mice expressing musclin-GFP plasmids. TA of 10 week-old male BALB/c C26-bearing mice were electroporated for 14 days with plasmids for GFP or musclin-GFP. In vivo transfection was monitored with specific probes by Q-PCR and gene expression of musclin and MuRF1 plotted as the fold change over controls (GFP-expressing muscles). *TBP* was used as housekeeping gene. Unpaired t test. *n* = 5–6.

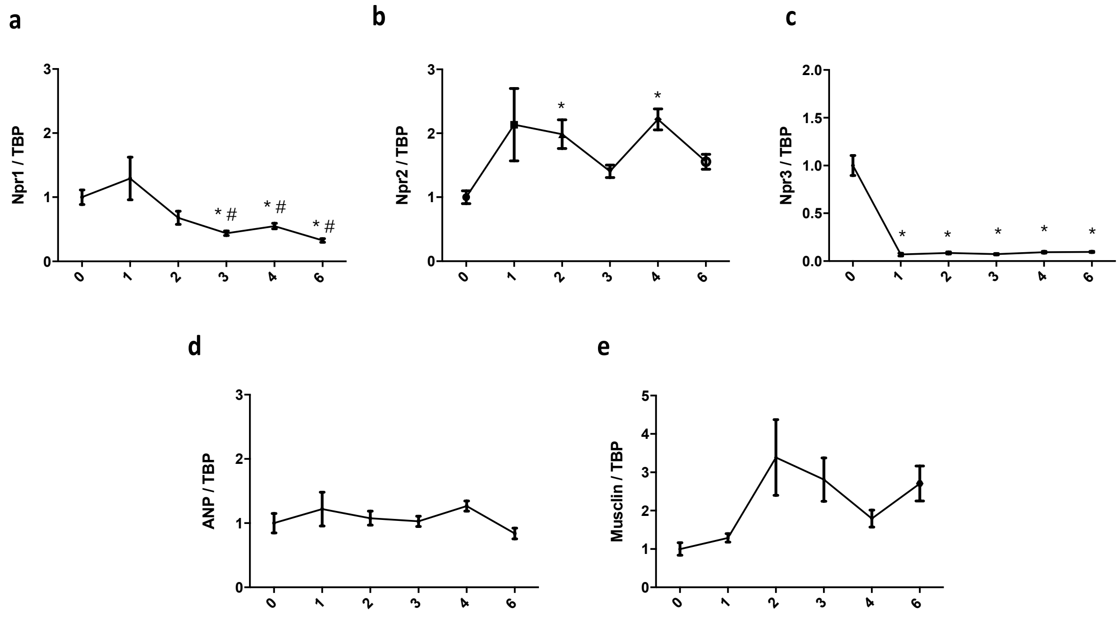

Figure S6. In differentiating myotubes, the expression of *Npr1* and *3* decreases and that of *Npr2* increases at times when ligands of these receptors, *ANP* or *musclin*, are mostly stable. Expression of *Npr1* (**a**), *Npr2* (**b**), *Npr3* (**c**), *ANP* (**d**) and *musclin* (**e**) are reported in myoblasts (day 0) and in myotubes at various days of differentiation. One-way ANOVA with repeated measures followed by Newman-Keuls post hoc test, * $p \leq 0.05$ vs day 0; # $p \leq 0.05$ vs day 1 (a), $n = 6$.

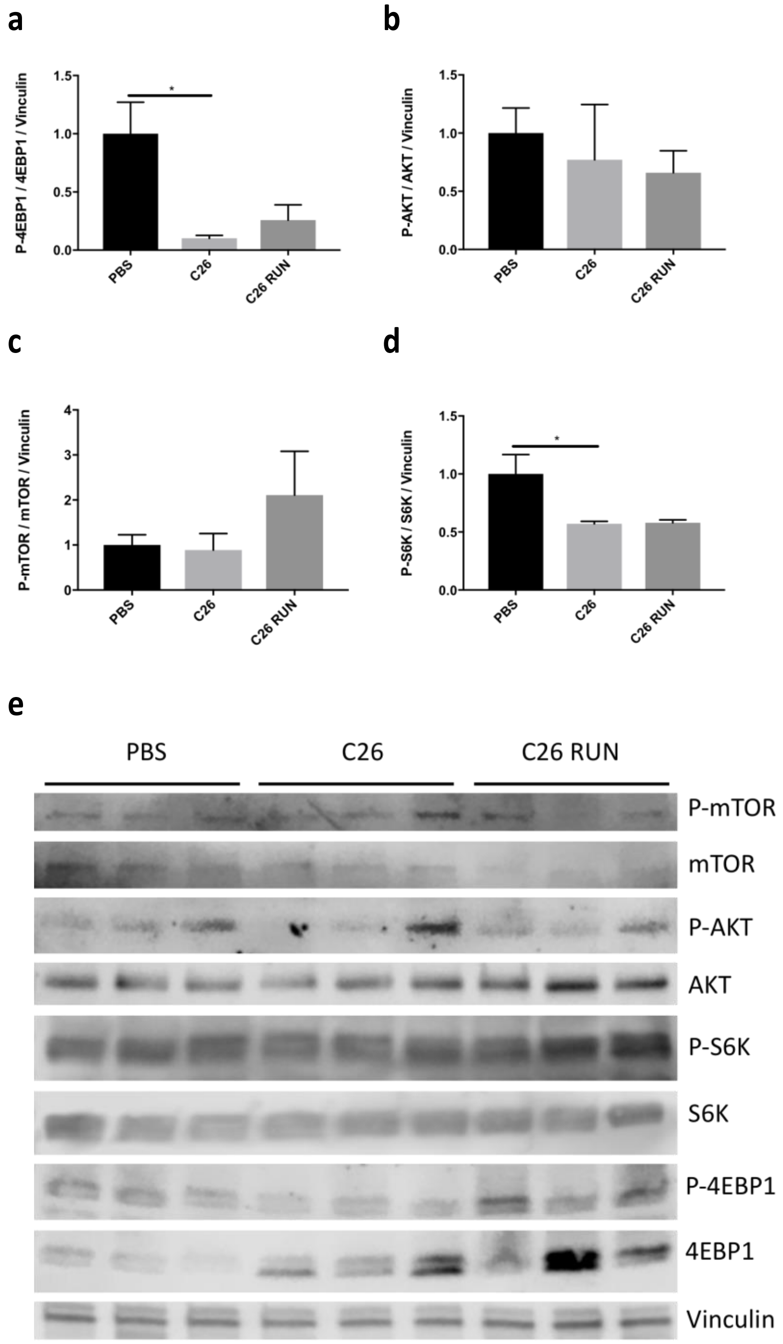

Figure S7. Five-day running does not induce AKT signaling in TA of C26-bearing mice. The densitometric analysis shows the ratio of P-4EBP1/4EBP1 (**a**), P-AKT/AKT (**b**), P-mTOR/mTOR (**c**), P-S6K/S6K (**d**), normalized for vinculin in gastrocnemius lysates from mice of the indicated group. Bands have been quantitated from blots shown in (**e**). The protein content of P-mTOR, mTOR, P-AKT, AKT, P-S6K, S6K, P-4EBP1 and 4EBP1 is blotted for lysates of gastrocnemius of PBS-injected, C26-bearing and C26-bearing mice subjected to run (**e**). Vinculin served as loading control. One-way ANOVA followed by Tukey's test, * $p \leq 0.05$, $n = 3$.

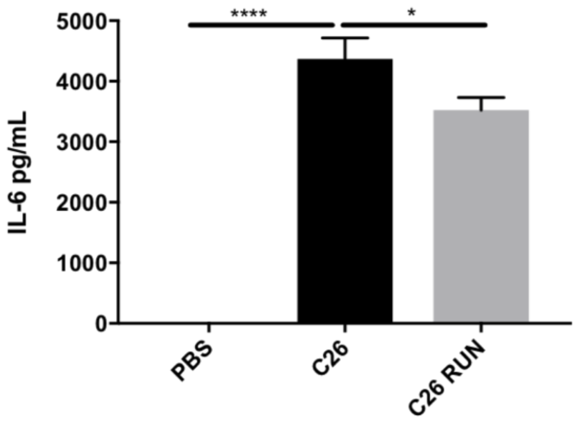

Figure S8. The plasma concentration of IL6 is increased in C26-bearing mice and partially restrained in five day-trained mice. One-way ANOVA followed by Tukey's test, * $p \leq 0.05$, **** $p \leq 0.0001$ $n = 8$–10.

Supplementary Methods

Polymerase Chain Reaction (PCR)

GFP adenovirus expression was measured in 10 µL of supernatant from infected myotubes by PCR using CMV primers, according to the following program: 95 °C 2 min, (95 °C 1 min, 65 °C 30 sec, 72 °C 1 min) × 40 cycles, 72 °C 10 min.

Mice and Tumor Model

RXF393 cells were injected (1.5 × 10^6 cells, subcutaneously) into the flank of six-to-eight-week-old female NCr-nu/nu mice (BW 19.8–22.4 g; Harlan Laboratories, Lesmo, Italy). Nude mice were maintained under specific pathogen-free conditions and handled using aseptic procedures.

Immunohistochemistry

Ten-µm thick cryosections of electroporated muscles were fixed with 4% paraformaldehyde and stained for nuclei with Hoechst (Sigma, St. Louis, MO, USA). Musclin antibody (1:30, sc-99096, Santa Cruz Biotechnology, Dallas, TX, USA) and Alexa 546-conjugated secondary antibody (A11035, Molecular Probes, Waltham, MA, USA) were used on muscle sections. Pictures of myotubes and muscle fibers were acquired with an Olympus Microscope IX71 (× 20 magnification, × 10 ocular lens) with Cell F (2.6 Build1210) imaging software for Life Science microscopy (Olympus Soft Imaging Solutions GmbH, Munster, Germany).

Immunoblotting

The antibodies used are as follows: 1:1000 anti-phospho-T389S6K (9205, Cell Signaling, Danvers, MA, USA), 1:1000 anti-P70 S6K (9202, Cell Signaling, Danvers, MA, USA), 1:1000 anti-phosphoSer65-4EBP1 (9451, Cell Signaling, Danvers, MA, USA), 1:1000 anti-4EBP1 (9452, Cell Signaling, Danvers, MA, USA), 1:1000 anti-AKT (BK9272s, Cell Signaling, Danvers, MA, USA), 1:1000 anti-phosphoSer473-AKT (BK9271S, Cell Signaling, Danvers, MA, USA), 1:1000 anti-phosphoSer2448mTOR (2971, Cell Signaling, Danvers, MA, USA), 1:1000 anti-mTOR (2972, Cell Signaling, Danvers, MA, USA).

ELISA

IL6 was measured with a Cayman Chemical ELISA kit (Cayman Chemical, East Ellsworth Road Ann Arbor, MI, USA). Diluted plasma samples and standards were applied to the ELISA plates, and incubated for 1 h at RT. Plates were then incubated with biotinylated antibody for 1h at RT. After four washes, plates were incubated with HRP-avidin 30 min at room temperature, then washed and incubated with the TMB Substrate for 30 min at RT. Optical density was detected at 450 nm. Samples were loaded in duplicates. The detection range of the kit is 23.4-1500 pg/mL and the sensitivity is 23 pg/mL.
